# Hydrogen Sulfide-Regulated NF-κB Signaling via Persulfidation: A Review

**DOI:** 10.3390/biom16081130

**Published:** 2026-08-03

**Authors:** Liang Xu, Keke Liang, Renjie Wang, Yanling Ta, Yongrun Yang, Jiaxing Wang, Xianxie Zhang, Yuguang Wang, Chengrong Xiao, Yihao Wang, Maoxing Li

**Affiliations:** 1 College of Pharmacy, Gansu University of Chinese Medicine, Lanzhou 730000, China; xuliang021026@163.com (L.X.); 18394248206@163.com (K.L.); tyl20250510@163.com (Y.T.); yangyongrun0207@163.com (Y.Y.); wjx010890@163.com (J.W.); zhangxianxie@163.com (X.Z.); 2 Academy of Military Medical Sciences, Beijing 100850, China; wangrenjiegy@163.com (R.W.); wyg79@139.com (Y.W.); xiaocr2026@163.com (C.X.); 3 College of Traditional Chinese Medicine, Tianjin University of Traditional Chinese Medicine, Tianjin 300193, China

**Keywords:** hydrogen sulfide (H_2_S), persulfidation (or persulfidation modification), nuclear factor kappa B (NF-κB) signaling pathway, inflammation, oxidative stress

## Abstract

Hydrogen sulfide (H_2_S) is an important endogenous gaseous signaling molecule that regulates diverse physiological and pathological processes through protein persulfidation. As a central regulator of inflammation and immune responses, NF-κB signaling is precisely controlled by post-translational modifications, and its dysregulation contributes to various inflammatory diseases. Recent studies reveal that H_2_S-mediated persulfidation is a key mechanism for fine-tuning NF-κB activity by targeting critical components, including p65, IKKβ, IκBα, and upstream regulators. Through site-specific S-sulfhydration, H_2_S modulates IKK activation, IκBα degradation, and p65 nuclear translocation, thereby limiting excessive NF-κB activation and inflammatory cytokine production. This review provides an integrated view of how endogenous H_2_S production and persulfidation-dependent signaling regulate inflammatory responses. Rather than simply summarizing individual pathways, we focus on the molecular mechanisms underlying H_2_S-mediated regulation of NF-κB-associated networks, including TLR4/NF-κB, PI3K/Akt/NF-κB, and MAPK/NF-κB pathways, and highlight its roles in oxidative stress, apoptosis, pyroptosis, and tissue repair. We further discuss current challenges in identifying persulfidation sites, understanding endogenous H_2_S regulation, and improving detection technologies. By proposing H_2_S as a precision modulator of inflammatory signaling, this review provides new insights into H_2_S biology and highlights future opportunities for developing targeted H_2_S-based therapeutic strategies.

## 1. Introduction

Hydrogen sulfide (H_2_S) is a colorless, irritating gas with a rotten egg odor. Traditionally, it has been regarded as a toxic and harmful gas [1]. However, recent molecular biology studies have confirmed that the body endogenously produces small amounts of H_2_S. Alongside nitric oxide (NO) and carbon monoxide (CO), H_2_S is now recognized as an important gaseous signaling molecule in the body, playing key regulatory roles in various physiological and pathological processes, including antioxidative stress [2], anti-inflammatory responses [3], inhibition of neuronal apoptosis [4], and protection of blood–brain barrier integrity [5]. Persulfidation is a special post-translational protein modification discovered after phosphorylation and acetylation. Its core mechanism involves the introduction of a sulfhydryl group (-SH) onto cysteine residues of proteins, forming a persulfide group (-SSH), which in turn regulates protein activity and function. This modification participates in intracellular signal transduction and the regulation of diverse biological processes, making it a hotspot in the field of protein modification [6].

Nuclear factor κB (NF-κB) is an important class of nuclear transcription factors. Its activation depends on the dissociation of the inhibitor of NF-κB (IκB), after which NF-κB translocates from the cytoplasm to the nucleus, binds to specific sites on target genes, and initiates transcription of downstream genes, thereby exerting its biological effects [7]. The NF-κB signaling pathway is central to the regulation of inflammatory responses and apoptosis. Previous studies have shown that endogenous H_2_S can participate in regulating key biological processes such as inflammation and apoptosis by S-sulfhydrating the RelA (p65) protein within the NF-κB pathway [8]. This review summarizes recent research progress on the molecular mechanisms and biological significance of H_2_S-mediated regulation of the NF-κB signaling pathway through persulfidation.

## 2. NF-κB Signaling Pathway

### 2.1. Overview of the NF-κB Signaling Pathway

Nuclear factor κB (NF-κB) is a ubiquitous and important nuclear transcription factor present in various cell types across almost all animal species. Its family members include five proteins: RelA (p65), RelB, c-Rel, p50/p105 (NF-κB1), and p52/p100 (NF-κB2). These typically exist as homo- or heterodimers, such as p50/p65 and p65/c-Rel, among which the p50/p65 heterodimer is the most common [9,10]. There are eight known inhibitors of NF-κB (IκBs): the classical IκB proteins (IκBα, IκBβ, IκBε), the NF-κB precursor proteins (p100, p105), and the nuclear IκBs (IκBζ, Bcl-3, and IκBNS). In most cells, these IκBs exist as inactive complexes in the cytoplasm and bind to NF-κB through specific ankyrin-repeat sequences at their C-termini [11,12]. Upstream of IκB, the IκB kinase (IKK) complex is a crucial kinase that activates the NF-κB system. IKK is a serine/threonine kinase complex composed of three subunits: IKKα (CHUK), IKKβ (IKBKB), and NEMO (IKBKG). It primarily functions by phosphorylating the IκBα protein bound to NF-κB in the cytoplasmic matrix, thereby releasing the dimeric protein and enhancing gene transcription [13,14].

### 2.2. Classical Activation Pathway

The aforementioned NF-κB dimers, IκB proteins, and IKKs constitute the NF-κB signaling pathway. Under resting conditions, NF-κB directly binds to IκB family inhibitory proteins and remains in an inactive state in the cytoplasm [15]. However, upon stimulation by signals such as tumor necrosis factor alpha (TNF-α), interleukin 1 (IL-1), bacterial lipopolysaccharide (LPS), or ultraviolet irradiation, RIP1 kinase is activated, facilitating the binding of these signals to their corresponding receptors to form complexes, which subsequently activate IKK. Activated IKKβ then phosphorylates IκBα at two amino acid residues, Ser32 and Ser36, at its N-terminus. Subsequently, phosphorylated IκBα undergoes ubiquitination and is hydrolyzed by the 26S proteasome, leading to its dissociation from NF-κB. This releases the NF-κB dimer, exposing its nuclear localization signal, which promotes the translocation (nuclear shift) of the NF-κB dimer from the cytoplasm to the nucleus. Once in the nucleus, the NF-κB dimer binds to specific sites on the promoters or enhancers of target genes, inducing their transcription [16,17,18,19]. There are hundreds of such target genes, including those encoding major proinflammatory cytokines, chemokines, IκBα, and RelB, thereby broadly participating in various biological processes such as immune responses, inflammation, cell proliferation, and apoptosis [20]. This is the classical pathway of NF-κB signaling activation.

### 2.3. Non-Canonical Activation Pathway

The NF-κB signaling pathway can also be activated through the non-canonical pathway. In the unstimulated state, NF-κB-inducing kinase (NIK) is recruited by TRAF2 and TRAF3 to cIAP1/2 (cellular inhibitor of apoptosis protein 1/2, cIAP1 and cIAP2), leading to K48-linked ubiquitination and subsequent degradation of NIK [21]. Activation of the non-canonical pathway primarily depends on stimulation by factors such as lymphotoxin-beta (LT-β), B-cell activating factor of the TNF family (BAFF), and cluster of differentiation 40 (CD40). Upon binding of these signaling factors to their cognate receptors, TRAF2 and TRAF3 are directly recruited to the receptors and degraded via the proteasome. Concurrently, the targets of cIAP1/2 are shifted, promoting NIK accumulation and subsequent phosphorylation, thereby activating IKKα. Activated IKKα then phosphorylates p100, which is partially ubiquitinated by the recruited SCFβTrCPβTrCP ubiquitin ligase complex, followed by proteolytic digestion of its C-terminal portion to release the N-terminal portion, i.e., p52. Subsequently, p52 forms RelB–p52 heterodimer with RelB and translocates into the nucleus to induce transcription of specific genes [22,23].

Under normal physiological conditions, the activation of the NF-κB signaling pathway is tightly regulated to maintain internal homeostasis. However, excessive activation or dysregulation of this pathway can lead to uncontrolled inflammatory responses and imbalance in cell proliferation and apoptosis. This is associated with the occurrence and progression of many diseases, including cardiovascular diseases, cancer, neurodegenerative diseases, and autoimmune diseases.

## 3. Endogenous Generation of Hydrogen Sulfide and Persulfidation Modification

### 3.1. Endogenous Hydrogen Sulfide Generation Pathways: Enzymatic and Non-Enzymatic Pathways

H_2_S has recently been recognized as a novel endogenous gaseous signaling molecule and has garnered widespread attention due to its anti-inflammatory, antioxidant, and cardiovascular protective effects. In mammals, the majority of H_2_S is generated via enzymatic pathways. The primary enzymes involved in H_2_S production are cystathionine-β-synthase (CBS), cystathionine-γ-lyase (CSE), and 3-mercaptopyruvate sulfurtransferase (3-MST) [24]. These three enzymes have distinct subcellular localizations and exhibit different expression levels across tissues. Among them, CBS is the major H_2_S synthase in the nervous system, with high expression in the brain, CSE is expressed in the cardiovascular system, as well as in the liver and kidneys, and 3-MST is mainly localized in mitochondria, with highest expression in the kidneys, followed by the liver and heart. Moreover, these three enzymes employ different catalytic mechanisms. CBS predominantly catalyzes the condensation of two cysteine molecules via β-replacement or the reaction of homocysteine with cysteine via γ-replacement to produce H_2_S. CSE can generate H_2_S directly through α-elimination, as well as via α,γ-elimination or γ-replacement. 3-MST, in coordination with cysteine aminotransferase (CAT), produces 3-mercaptopyruvate, which then forms a persulfide intermediate and subsequently reacts with endogenous reducing agents such as thioredoxin to generate H_2_S [25,26,27]. Notably, a small amount of H_2_S is also produced through non-enzymatic reactions, for example, by sulfate- and sulfite-reducing bacteria [28]. This reaction occurs predominantly in red blood cells. Beyond H_2_S biosynthesis itself, the gas also engages in sophisticated cross talk with other sulfur-containing metabolic networks. Substantial evidence indicates that H_2_S does not regulate GSH metabolism through a single linear pathway, but rather acts at multiple interconnected levels. H_2_S intersects with GSH biosynthesis through the transsulfuration pathway. H_2_S is produced from L-cysteine by cystathionine β-synthase (CBS) and cystathionine γ-lyase (CSE), both of which also generate cysteine. Thus, H_2_S production and cysteine availability are metabolically coupled. Moreover, cysteine derived from N-acetylcysteine (NAC) serves as a substrate for both GSH synthesis and H_2_S production, suggesting a dynamic interaction between these two sulfur-containing metabolites. At the redox cycling level, H_2_S maintains the GSH/GSSG ratio by enhancing the activity of glutathione reductase (GR), promoting the regeneration of GSSG to GSH. This effect is particularly important under oxidative stress conditions, where maintaining the GSH–GSSG balance is critical for cellular redox homeostasis [29,30]. Furthermore, H_2_S plays important roles in antioxidative stress, apoptosis, inflammatory responses, and cardiovascular diseases, with persulfidation mediated by H_2_S being the primary mechanism underlying its biological effects [31].

In addition, CSE is not constitutively highly expressed in humans. Rather, its expression is upregulated upon stimulation, when transcription factor SP1 binds to the promoter region of CSE, leading to a significant increase in CSE mRNA. Elevated CSE protein levels subsequently result in increased intracellular H_2_S production. In the NF-κB signaling pathway, H_2_S acts as a nucleophile to attach an additional sulfur atom to the thiol group (-SH) of p65-Cys38, generating a persulfide group (-SSH). This modification induces a conformational change in the N-terminal region of p65, exposing the binding site for coactivators. The persulfidated p65 then recruits ribosomal protein S3 (RPS3), ultimately protecting the organism from injury. In CSE-knockdown models, however, this cascade of events fails to occur [8,32].

Notably, studies have shown that 3-mercaptopyruvate sulfurtransferase (3-MST) itself functions as a protein persulfidase. It specifically recognizes 3-mercaptopyruvate (3-MPA) and attacks the sulfur atom in 3-MPA, initiating a β-elimination reaction to generate the 3-MST-SSH intermediate. In this intermediate, the outer sulfur atom forms an unstable persulfide bond (−S−S−) with the sulfur atom at the enzyme’s active site. Subsequently, the thiol group of a cysteine residue on the target protein acts as a nucleophile, directly attacking the outer sulfur atom of 3-MST-SSH, resulting in the transfer of the outer sulfur atom to the target protein and formation of the target protein persulfide. The active-site cysteine of 3-MST is restored to its free thiol state, enabling the next round of catalysis. However, due to steric constraints, the inner sulfur atom of 3-MST-SSH is not readily attacked by nucleophiles, and thus this reaction rarely occurs [33].

In addition, quantification methods for H_2_S include UV-vis spectrophotometry, chromatography, and fluorescence probe methods, etc. Enzyme activity detection methods include LC-MS/MS and fluorescence probe methods, etc. [29], as shown in Table 1 and Table 2.

However, for exogenous H_2_S, each donor exhibits distinct release profiles. In the studies by Yanxi Liu [54], Tae Jun Kim [55], and colleagues, it was found that SFN and GYY4137 show similar H_2_S release kinetics at their respective IC_50_ concentrations—namely a rapid release during the first 30 min, followed by a plateau. In addition, Na_2_S and NaHS release H_2_S rapidly and in large amounts in PBS; however, their release is significantly attenuated in serum-containing medium due to protein binding. In contrast, DATS and DADS show minimal H_2_S release in PBS, but their release increases rapidly upon the addition of glutathione (GSH).

### 3.2. Mechanisms of Persulfidation Modification

**Figure 1:**
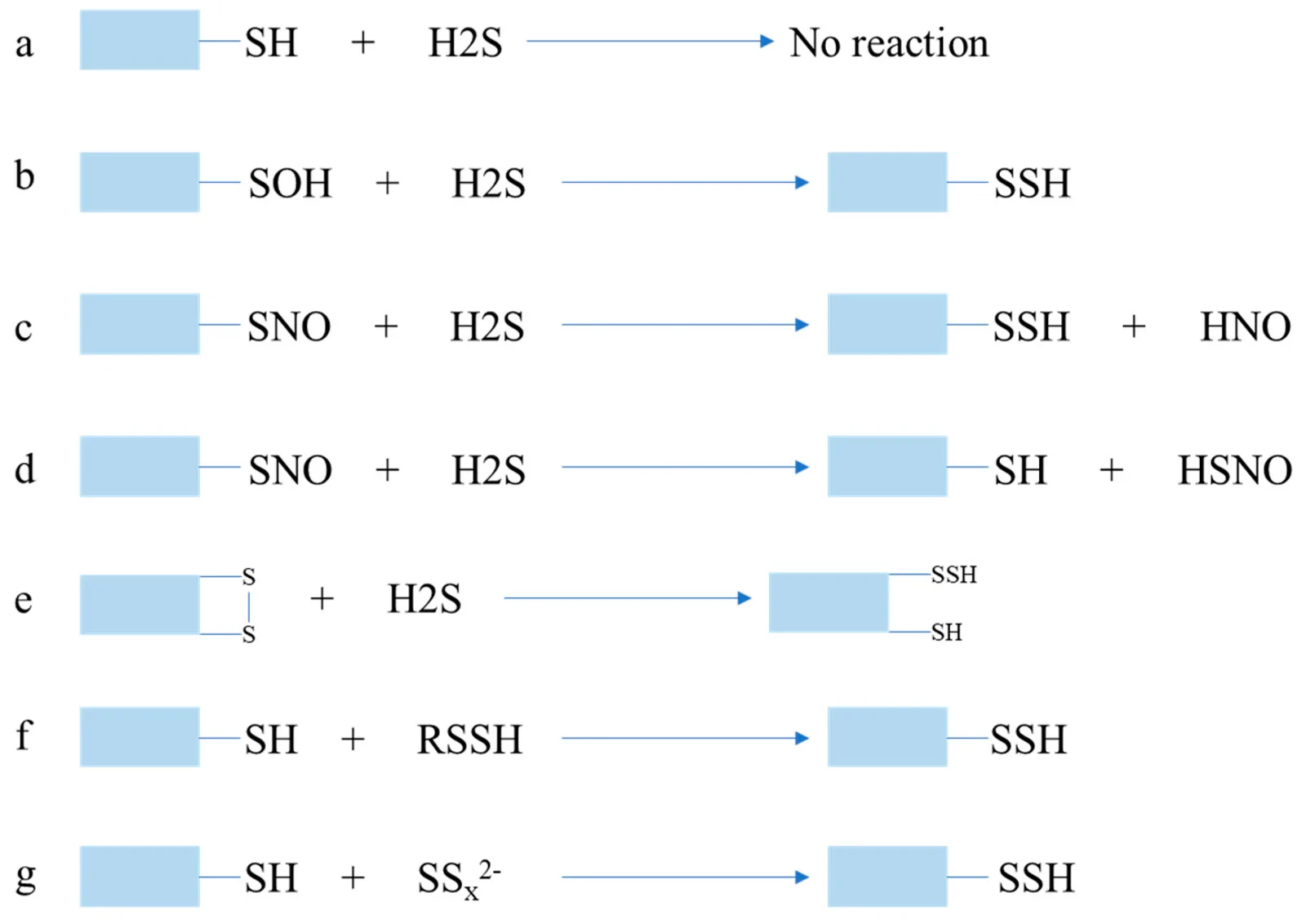
The formation process of persulfidation: (**a**) H_2_S cannot directly react with thiol groups (-SH); (**b**) H_2_S can react with the sulfenic acid form of cysteine (Cys-SOH) to generate persulfidation; (**c**,**d**) H_2_S can react with S-nitrosated cysteine to generate nitroxyl (HNO) or thionitrous acid (HSNO); (**e**) H_2_S can react with cysteine disulfides (−S−S−) to generate persulfidation; (**f**) persulfides (RSSH) can act as carriers to undergo transpersulfidation reactions with RSH groups; (**g**) oxidized sulfur species such as polysulfides (Sx2^−^x2^−^) can react with RSH to generate persulfidation.

Hydrogen sulfide-mediated persulfidation is a novel post-translational protein modification that plays a critical role in maintaining cellular homeostasis and signal transduction [56]. The mechanisms by which H_2_S induces persulfidation include the following: (1) due to thermodynamic constraints, H_2_S cannot directly react with the cysteine thiol group (RSH) of proteins, but it can react with the sulfenic acid form of cysteine (Cys-SOH) to produce persulfidation; (2) under different environmental conditions, H_2_S can react with S-nitrosated cysteine to generate nitroxyl (HNO) or thionitrous acid (HSNO); (3) H_2_S reacts with cysteine disulfides (−S−S−) to form S-sulfhydrated products; (4) persulfides (RSSH) can act as carriers and participate in transpersulfidation reactions through acid–base motifs spatially close to RSH groups; and (5) persulfidation can also be formed via reactions between oxidized sulfur species such as polysulfides (Sx2^−^x2^−^) and RSH groups (Figure 1) [57].

### 3.3. Detection Methods for Persulfidation

Currently, the main detection methods for persulfidation include the maleimide assay, biotin-switch assay, tag-switch assay, cysteine labeling assay, mass spectrometry, and diketone method, among others.

#### 3.3.1. Maleimide Assay

This method was proposed by Professor Snyder and his team at Johns Hopkins University in 2012. It is a relatively sensitive technique for detecting protein persulfidation. This approach uses an Alexa Fluor 680-conjugated C2 maleimide fluorescent probe to label the thiol groups of cysteine residues. The sample is then treated with the reducing agent dithiothreitol (DTT), which selectively cleaves disulfide bonds and removes the -SH group from -SSH, leading to a decrease in fluorescence signal. The fluorescence signal intensity can be assessed by separating proteins via gel electrophoresis and transferring them onto a PVDF membrane using transfer or dot blotting. The red fluorescent bands on the PVDF membrane are detected using a gel imaging system, allowing observation of changes in fluorescence signal intensity. A decrease in fluorescence signal indicates persulfidation of the target protein by H_2_S. However, this method cannot be used for large-scale proteomic analysis [58].

In addition, another maleimide-based labeling method is the biotin-thiol assay (BTA). First, biotin–polyethylene glycol–maleimide is used to simultaneously label both thiol and persulfide sites on proteins. Second, the proteins are enzymatically digested, and all biotinylated peptides are captured using streptavidin beads. Finally, the reducing agent DTT is used to selectively cleave persulfide bonds, thereby eluting only the sulfhydrated peptides for subsequent mass spectrometry identification. However, it should be noted that high concentrations of maleimide can disrupt polysulfide chains, and the procedure is cumbersome [59].

#### 3.3.2. Biotin-Switch Assay

The biotin-switch assay, proposed by Mustafa and colleagues, was the original method for detecting S-sulfhydrated proteins. This method is a modification of an earlier approach used for detecting S-nitrosylation of cysteine thiols. Briefly, the thiol-blocking agent methyl methanethiosulfonate (MMTS) is first reacted with -SH groups. The MMTS is then removed with acetone, and -SSH groups are labeled with biotin-HPDP (N-(6-(biotinamido)hexyl)-3′-(2′-pyridyldithio)-propionamide) in dimethyl sulfoxide (biotin-HPDP does not react with MMTS). Biotinylated proteins are then captured and pulled down using streptavidin–agarose beads, followed by washing with HENS buffer. Finally, the biotinylated proteins are eluted with SDS-PAGE sample buffer and analyzed by Western blotting [60]. A limitation of this method is that MMTS cannot distinguish between -SH and -SSH and blocks both, preventing biotin-HPDP from labeling -SSH. Consequently, this method suffers from low specificity and a high false-positive rate.

#### 3.3.3. Tag-Switch Assay

Zhang and colleagues developed a method termed the “tag-switch assay” for detecting protein persulfidation. This group used methylsulfonyl benzothiazole (MSBT) and its more water-soluble analog MSBT-A to react specifically with sulfhydrated sites (-SSH), generating mixed aromatic disulfides that selectively block both free thiols and sulfhydrated sites. Subsequently, a nucleophilic biotinylated cyanoacetate derivative reacts with the disulfide bonds, and the labeled proteins are specifically captured by streptavidin–agarose beads via biotin. Finally, the proteins are analyzed by Western blotting to quantitatively assess persulfidation levels.

In addition, this group developed cyanoacetate derivatives bearing a fluorescent BODIPY moiety (CN-BOT) or a Cy3 dye (CN-Cy3) to detect sulfhydrated proteins by fluorescence microscopy or gel staining, thereby enhancing detection sensitivity. However, CN-Cy3 is difficult to wash out from fixed cells; therefore, CN-BOT is used for in situ detection of protein sulfhydration, while CN-Cy3 is used for detecting protein sulfhydration in cell lysates [61]. A limitation of this method is that the MSBT reagent cannot penetrate cell membranes and thus cannot be used for real-time detection in live, intact cells: it is only applicable to cell lysates or fixed cells.

#### 3.3.4. Cysteine Labeling Assay

This method was proposed by Krishnan and colleagues in 2011. First, iodoacetic acid (IAA) is used to label all -SH and -SSH groups. Then, DTT is used to reduce -SSH to -SH. Finally, an iodoacetamide-linked biotin probe (IAP) is used for labeling [62]. A limitation of this method is that it cannot distinguish -SSH from other reducible modifications such as S-nitrosylation.

#### 3.3.5. Mass Spectrometry

Longen and colleagues developed a mass spectrometry-based method for detecting protein persulfidation in 2016. First, the team treated cells with trichloroacetic acid (TCA) to achieve complete cell lysis and protein denaturation. Next, a biotinylation reagent called iodoacetyl-PEG2-biotin (IAMBio) was used to simultaneously label both -SH and -SSH groups on all proteins. The labeled proteins were then digested with trypsin, enriched using streptavidin–agarose beads, and eluted using tris(2-carboxyethyl)phosphine (TCEP) to release -SSH-derived peptides. Finally, the samples were analyzed by LC-MS/MS [63].

#### 3.3.6. Diketone Method

The diketone method is a novel approach for detecting protein persulfidation based on dimedone developed by Zivanovic and colleagues in 2019. First, they used a new labeling agent, 4-chloro-7-nitrobenzofurazan (NBF-Cl), which reacts with thiols (-SH), persulfides (-SSH), sulfenic acids, and amines. Then, a biotinylated dimedone probe (DCP-Bio1) was employed for “tag switching,” allowing -SSH to be readily captured by streptavidin magnetic beads and subsequently detected [64]. The limitations of this method include its relatively complex procedure and the need to consider the membrane permeability of the probes.

## 4. Biological Effects of H_2_S-Mediated Persulfidation of NF-κB

### 4.1. Biological Effects of H_2_S-Mediated Persulfidation of TLR4/NF-κB

Toll-like receptor 4 (TLR4) is a transmembrane protein and a member of the pattern recognition receptor family, which is indispensable for the activation of innate and adaptive immune responses [65]. It consists of an extracellular domain, a transmembrane domain, and a cytoplasmic Toll–interleukin 1 receptor (TIR) domain. The extracellular domain contains multiple leucine-rich repeat (LRR) motifs, each composed of 20–29 residues, providing specific sites for ligand interactions [66]. The transmembrane domain primarily maintains the topology of TLR4. The TIR domain plays a critical role in transducing extracellular signals into the intracellular space. Upon exposure to external stimuli, TLR4 recognizes and binds to these stimuli, subsequently recruiting the downstream adaptor protein MyD88, which further activates the IKK complex, promotes IκB phosphorylation and ubiquitination-dependent degradation, and ultimately enables NF-κB dimers to translocate into the nucleus to initiate gene transcription [67].

In recent years, numerous studies have demonstrated that H_2_S-mediated persulfidation of TLR4/NF-κB plays a significant role in regulating immune and inflammatory responses. In a study by Sun et al. [68], a lipopolysaccharide (LPS)-induced inflammatory model in bovine mammary epithelial cells (MAC-T) showed decreased cell viability and upregulated expression of TLR4 and NF-κB, effects that were reversed by NaHS treatment. In a Parkinson’s disease model, Cao et al. [69] found decreased cell viability, increased apoptosis rate, elevated levels of proinflammatory cytokines, and increased protein expression of TLR4, p-IκBα, p-NF-κB p65, and iNOS. Exogenous H_2_S treatment suppressed these increases, reduced proinflammatory cytokine release, and improved cell viability. Furthermore, in an LPS-induced infectious preterm birth model [70], NaHS administration reduced ASC and caspase 1 activity and downregulated TLR4 and NF-κB expression, suggesting that exogenous H_2_S alleviates LPS-induced inflammatory responses, potentially involving over-vulcanization modification. Liang et al. [71] established a salt-sensitive hypertensive rat model and found that protein levels of TLR4, MyD88, p-NF-κB p65, and p-IKKβ were significantly higher in the model group compared with the control group. Exogenous H_2_S inhibited the activation of the TLR4/MyD88/NF-κB signaling pathway, alleviated central neuroinflammation, subsequently reduced peripheral sympathetic nerve activity, and effectively lowered blood pressure. In a chronic obstructive pulmonary disease (COPD) model established by cigarette smoke exposure, Wang et al. [72] found that the expression levels of GSDMD-N, cleaved caspase 1, LDH, TLR4, and NF-κB were significantly increased compared with controls, and NaHS treatment reversed these changes. Additionally, Abdel-Latif [73] used CP to induce a rat hepatotoxicity model and observed decreased GSH levels, increased TBARS and NOx levels, and significantly increased expression of TLR2, TLR4, p-JNK, and NF-κB. NaHS treatment inhibited these changes. Similarly, in an alcohol-induced fatty liver model established by ethanol gavage [74], the NaHS-treated group showed significantly improved liver function indices and downregulated protein expression of TLR4 and NF-κB.

**Figure 2:**
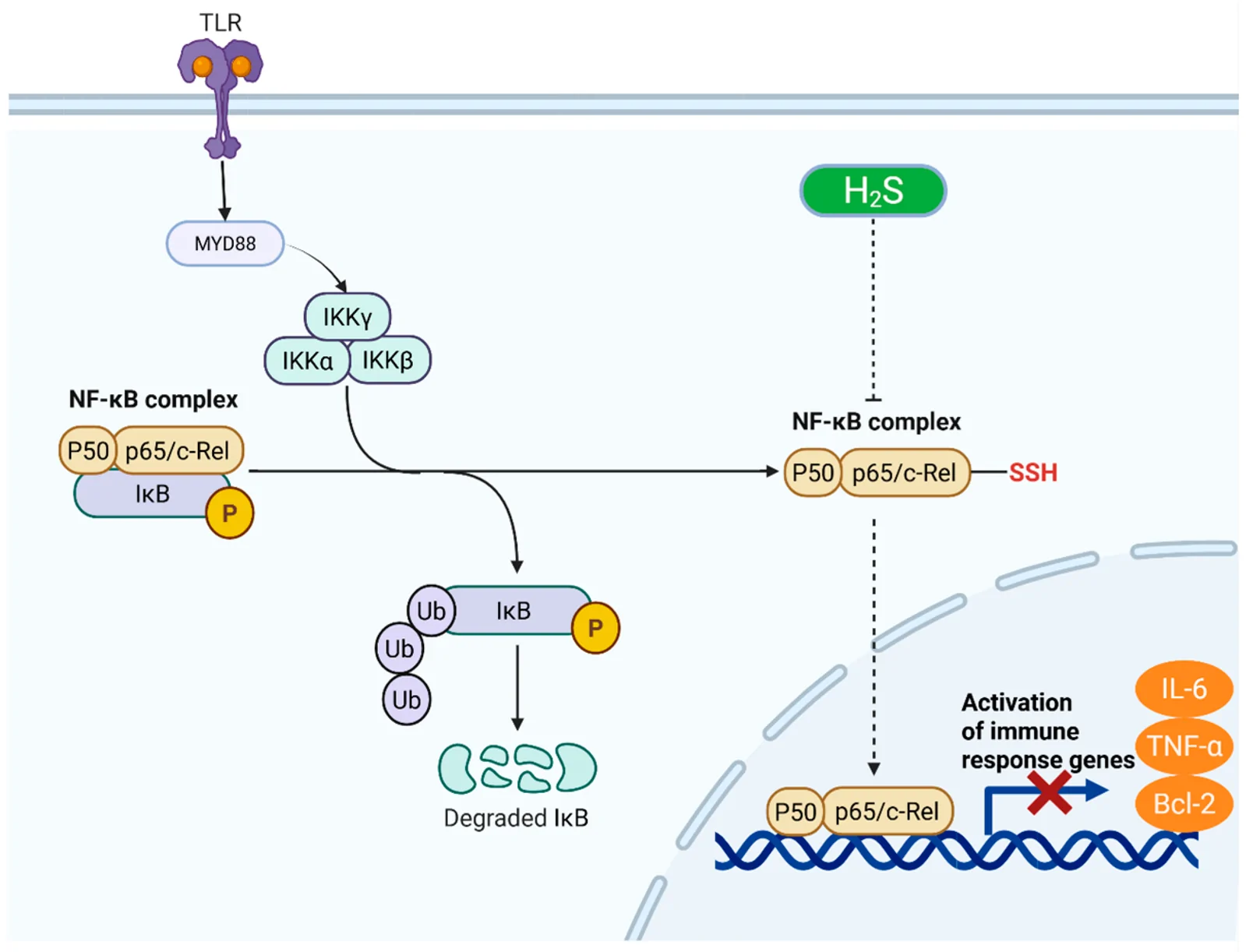
H_2_S regulates the TLR4/NF-κB signaling pathway through persulfidation modification. Abbreviations: NF-κB: nuclear factor κB; IκB: inhibitor of NF-κB; TLR: Toll-like receptor. Created in BioRender. Liang, K. (2026) https://BioRender.com/0kc3rdt (accessed on 22 July 2026).

Taken together, current studies both domestically and internationally have confirmed the regulatory role of H_2_S in the TLR4/NF-κB signaling pathway across various disease models and at different cellular levels. The core mechanism consistently points to persulfidation modification: H_2_S can S-sulfhydrate key proteins in the TLR4/NF-κB pathway (such as TLR4, RelA/p65, and IKK), thereby inhibiting excessive activation of the pathway, alleviating overt inflammatory responses, suppressing aberrant apoptosis, and exerting tissue-protective effects. However, existing studies still have certain limitations. Most research has focused on the intervention effects of exogenous H_2_S, whereas the regulatory mechanisms of endogenous H_2_S-mediated persulfidation remain insufficiently explored. Moreover, the precise modification sites on key proteins within the TLR4/NF-κB pathway, modification efficiency, and cross talk with other post-translational modifications require further in-depth investigation. In the future, with continued advances in research technologies, a deeper understanding of the molecular mechanisms of H_2_S-mediated persulfidation of the TLR4/NF-κB pathway will provide new insights and potential therapeutic targets for the prevention and treatment of various diseases, including inflammation-related diseases, neurodegenerative disorders, and cardiovascular diseases (Figure 2).

### 4.2. Biological Effects of H_2_S-Mediated Persulfidation of PI3K/Akt/NF-κB

Phosphoinositide 3-kinase (PI3K) is a member of the lipid kinase family. PI3K is classified into three classes (I, II, and III) based on its structure and substrate specificity. Class I PI3K is the most extensively studied and is further divided into the IA and IB subclasses. Class IA PI3K is a heterodimer composed of a catalytic subunit p110 (p110α, p110β, and p110γ) and a regulatory subunit p85 (p85α, p85β, and p85γ). Class IB PI3K consists of a regulatory subunit p101 and a catalytic subunit p110γ [75,76]. Protein kinase B (Akt) is a member of the serine/threonine kinase family and is a key downstream target of PI3K. It consists of three conserved domains: an N-terminal pleckstrin homology (PH) domain, a central kinase catalytic domain, and a C-terminal hydrophobic motif (HM). Akt has three isoforms: PKBα (Akt1), PKBβ (Akt2), and PKBγ (Akt3). Each isoform exhibits different tissue expression levels. Akt1 is widely expressed across various tissues, whereas Akt2 is highly expressed in insulin-sensitive tissues, and Akt3 is specifically found in the brain, lung, heart, kidney, testis, and skeletal muscle [77,78].

PI3K can be activated via receptor tyrosine kinases (RTKs), G protein-coupled receptors (GPCRs), and Ras-related small G proteins. Activated PI3K converts phosphatidylinositol 4,5-bisphosphate (PIP2) on the cell membrane into the second messenger phosphatidylinositol 3,4,5-trisphosphate (PIP3). The generated PIP3 specifically binds to the PH domain of downstream Akt, promoting the translocation of Akt and 3-phosphoinositide-dependent protein kinase 1 (PDK1) to the cell membrane. Subsequently, PDK1 and mammalian target of rapamycin complex 2 (mTORC2) catalyze the phosphorylation of Akt at Thr-308 and Ser-473, respectively, thereby activating Akt. The activated PI3K/Akt pathway stimulates the IKK complex, leading to IκB phosphorylation and degradation, promoting NF-κB nuclear translocation, and consequently initiating gene transcription [79].

It is worth noting the substantial evidence that H_2_S-mediated persulfidation of the PI3K–Akt–NF-κB axis plays an important role in maintaining body homeostasis. In a sepsis model, Liu et al. [80] found that exogenous H_2_S administration increased PI3K/Akt phosphorylation and decreased the expression of NF-κB, Bax, and caspase 3. In a septic acute lung injury model [81], exogenous H_2_S improved survival and reduced lung injury in septic mice. Moreover, compared with the model group, GYY4137 decreased p-Akt and p-NF-κB p65 protein expression levels in a dose-dependent manner, a process potentially related to persulfidation modification. Wang et al. [82] established an LPS-induced inflammatory model in bovine mammary epithelial cells and found that exogenous H_2_S activated the PI3K/Akt/NF-κB signaling pathway and regulated LPS-induced inflammation and apoptosis in a concentration-dependent manner. In a portal hypertension model [83], exogenous H_2_S administration led to significantly increased p-Akt and NF-κB levels, decreased IκBα and Bcl-2 levels, and increased apoptosis compared with the model group. In addition, research [84] found that H_2_S promoted LPS-induced osteoblast apoptosis and exacerbated inhibition of osteogenic differentiation by inhibiting the AKT/NF-κB signaling pathway and enhancing the FAS/FASL-mediated death receptor pathway. Furthermore, in an acute pancreatitis model induced by caerulein, Fu et al. [85] found that NaHS treatment inhibited the PI3K/AKT-NF-κB signaling pathway and downregulated proinflammatory cytokine levels, thereby alleviating inflammatory damage to pancreatic acinar cells. In a subsequent rat severe pancreatitis model, Rao et al. [86] observed increased p-Akt expression, decreased IκBα expression, enhanced NF-κB activity, and aggravated inflammation in the model group. Exogenous H_2_S (NaHS treatment) alleviated pancreatic inflammatory damage by inhibiting the PI3K/AKT/NF-κB signaling pathway and downregulating proinflammatory cytokine expression. In a myocardial injury model of sleep apnea syndrome induced by chronic intermittent hypoxia, Zheng et al. [87] found that chronic intermittent hypoxia activated the PI3K/AKT/NF-κB pathway, increased apoptosis and oxidative stress (OS) levels, and exacerbated pathological damage, whereas NaHS reversed these effects. Zheng et al. [88] used a hypoxia–reoxygenation model in mouse atrial cardiomyocytes and found that the model group exhibited reduced cell numbers, increased malondialdehyde (MDA) levels and NF-κB protein expression, and decreased superoxide dismutase (SOD) activity. Pretreatment with NaHS reversed these effects and reduced apoptosis.

**Figure 3:**
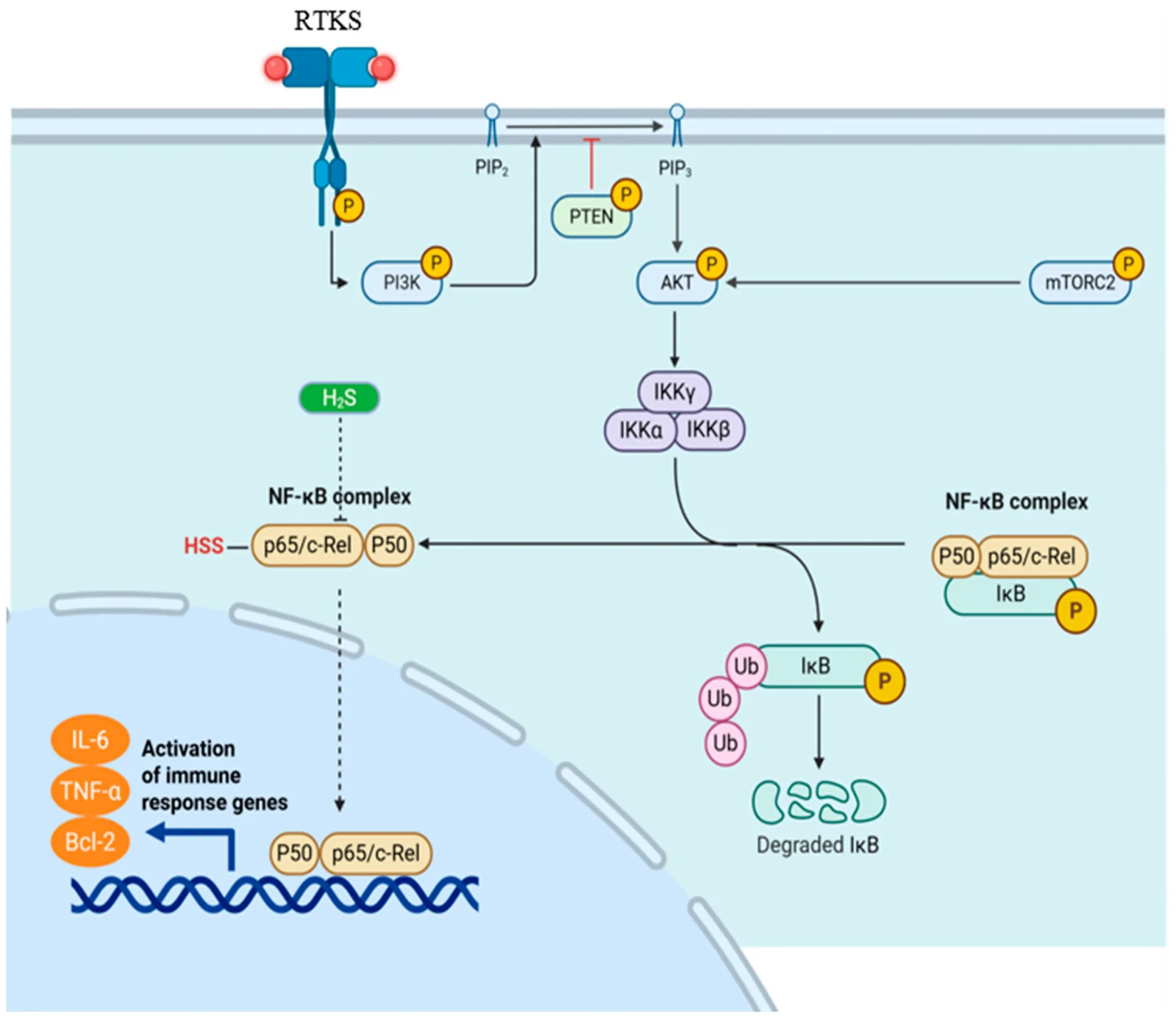
H_2_S regulates the PI3K/Akt/NF-κB signaling pathway through persulfidation modification. Abbreviations: NF-κB: nuclear factor κB; IκB: inhibitor of NF-κB; PI3K: phosphoinositide 3-kinase; AKT: protein kinase B. Created in BioRender. Liang, K. (2026) https://BioRender.com/w2qn7za (accessed on 22 July 2026).

Collectively, these studies demonstrate that H_2_S plays an important role in maintaining body homeostasis by mediating persulfidation modification of key proteins in the PI3K/Akt/NF-κB pathway, with its regulatory effects being disease model-dependent. However, current research has notable limitations. Most studies have focused on the intervention effects of exogenous H_2_S, whereas the regulatory mechanisms of endogenous H_2_S-mediated persulfidation of this pathway remain poorly understood. The specific key proteins within the PI3K/Akt/NF-κB pathway that are modified by H_2_S, the precise modification sites, and the modification efficiency have not yet been identified. Moreover, the reasons underlying the bidirectional regulatory effects of H_2_S in different disease models remain insufficiently explored. Additionally, the cross-regulatory relationships between H_2_S-mediated persulfidation and other post-translational modifications such as phosphorylation and acetylation within this pathway have not been elucidated. Furthermore, most existing studies are limited to cellular and animal models, with insufficient clinical evidence. These research gaps restrict a comprehensive understanding of the molecular mechanisms by which H_2_S regulates this pathway and hinder the development and translation of targeted therapeutic strategies based on this modification mechanism (Figure 3).

### 4.3. Biological Effects of H_2_S-Mediated Persulfidation of MAPK/NF-κB

Mitogen-activated protein kinases (MAPKs) play a critical role in transducing signals from the cell surface to the nucleus. To date, 17 MAPK members have been identified in mammals, which are classified into seven subgroups. Among these, the four conventional subgroups are extracellular signal-regulated kinase 1/2 (ERK1/2), c-Jun N-terminal kinase (JNK), p38 MAPK, and ERK5. The remaining three non-conventional subgroups are ERK3/4, ERK7/8, and Nemo-like kinase (NLK) [89].

The classical MAPK activation pathway strictly follows a three-tiered kinase cascade: MAPK kinase kinase (MAPKKK) → MAPK kinase (MAPKK) → MAPK [90]. Notably, the four conventional subgroups within the classical MAPK pathway can be activated by distinct stimuli, forming different signaling cascades and activating various transcription factors to exert diverse biological effects. Among them, ERK is generally associated with cell growth, whereas JNK and p38 are linked to inflammatory stress or infection [91]. In contrast, the activation pathways of atypical MAPKs do not adhere to the classical three-tiered kinase cascade, but are activated through various mechanisms, including monophosphorylation, autophosphorylation, protein-protein interactions, and ubiquitination. Upon MAPK pathway activation, extracellular signals are transduced into the cell, promoting IκB phosphorylation and ubiquitination-dependent degradation, which allows NF-κB dimers to translocate to the nucleus, activate the NF-κB pathway and promote gene transcription [92,93].

H_2_S plays an important role in protecting the body by S-sulfhydrating the MAPK/NF-κB signaling pathway. In chronic renal failure (CRF) [94], the protein levels of p-ERK, p-JNK, p-p38, and NF-κB were significantly elevated and NaHS intervention suppressed these increases, potentially involving over-vulcanization modification. Furthermore, in an LPS-induced inflammatory model of rat skeletal muscle cells established by Zhang et al. [95], H_2_S was found to inhibit the phosphorylation of ERK, JNK, and p38 and to reduce NF-κB protein expression. In a study by Chi [96], male broilers exposed to H_2_S gas over different periods showed increased mRNA and phosphorylated protein expression levels of JNK, ERK, and p38, along with increased IKKα/β and p65 expression, suggesting that H_2_S exposure causes cumulative damage to the avian immune system following long-term low-dose H_2_S exposure. In an alloxan-induced diabetic model, Song et al. [97] detected significantly higher mRNA expression of p38 MAPK and NF-κB p65 in the diabetic group compared with the control group. NaHS treatment reduced these expression levels, with more pronounced reduction in the high-dose group, suggesting that H_2_S ameliorates diabetic myocardial fibrosis. Li [98] induced NIH3T3 cells to simulate an in vitro pulmonary fibrosis microenvironment and found that exogenous H_2_S markedly inhibited TGF β1-induced cell proliferation. In addition, NaHS suppressed the activation of the p38 MAPK/NF-κB signaling pathway induced by TGF β1 and downregulated the transcription of the p38 MAPK and NF-κB genes.

**Figure 4:**
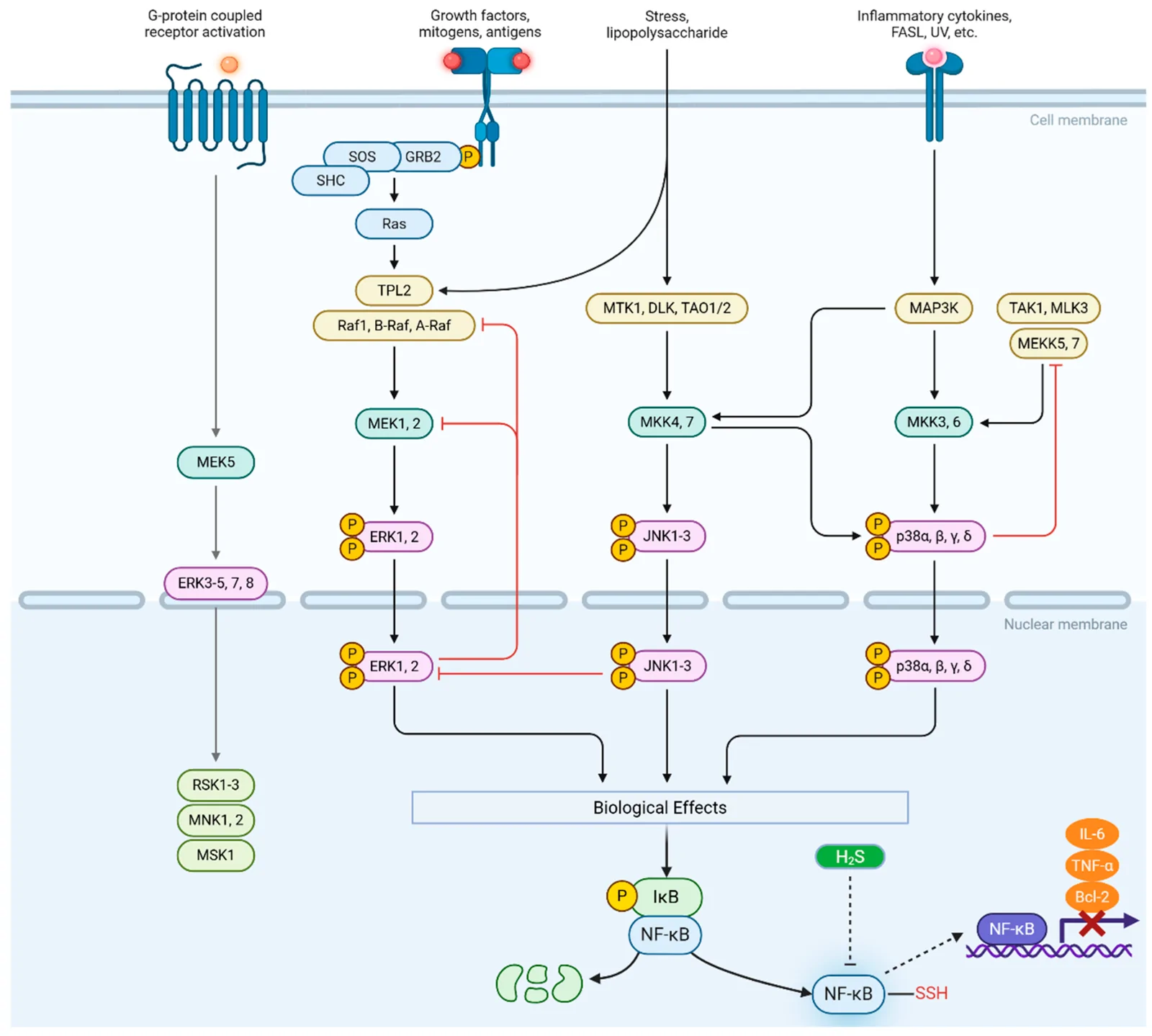
H_2_S regulates the MAPK/NF-κB signaling pathway through persulfidation modification. Abbreviations: NF-κB: nuclear factor κB; IκB: inhibitor of NF-κB. Created in BioRender. Liang, K. (2026) https://BioRender.com/w2qn7za (accessed on 22 July 2026).

A review of current related studies reveals that most remain at the level of observing pathway protein expression and pathological phenotypes, only preliminarily suggesting that the effects rely on persulfidation modification. The specific modification targets and cysteine modification sites within the MAPK/NF-κB pathway have not yet been identified. The intrinsic molecular mechanisms underlying the opposite biological effects of H_2_S at different concentrations and durations of exposure remain insufficiently explained. Moreover, there is a lack of systematic mechanistic validation and in vivo targeted intervention evidence. The relevant pathway networks and upstream/downstream cross-regulatory relationships still require further in-depth investigation and clarification (Figure 4).

### 4.4. Biological Effects of H_2_S-Mediated Persulfidation of STAT3/NF-κB

The signal transducer and activator of transcription (STAT) family consists of seven members: STAT1, STAT2, STAT3, STAT4, STAT5a, STAT5b, and STAT6. STAT family proteins respond to various extracellular growth factors and cytokines and are a class of SH2-containing signaling molecules that bind to phosphorylated tyrosine residues [99]. Among them, STAT3 is the most extensively studied. In the resting state, STAT3 exists in an inactive form in the cytoplasm. Upon stimulation by extracellular cytokines or growth factors, these factors bind to their cognate receptors, inducing conformational changes in the receptors, which in turn activate the non-receptor tyrosine kinases Janus kinases (JAK1, JAK2, JAK3, TYK2). The activated JAKs phosphorylate the receptors, creating binding sites for the SH2 domain of STAT3, promoting STAT3 binding, followed by its phosphorylation at Tyr-705 by JAKs. Subsequently, phosphorylated STAT3 dimerizes and translocates into the nucleus to initiate gene transcription [100]. STAT3 and NF-κB are closely interconnected. Activation of NF-κB can lead to STAT3 phosphorylation and nuclear translocation. Conversely, persistently activated STAT3 can prolong the nuclear retention of NF-κB, thereby maintaining its activity. Furthermore, they can even share or compete for overlapping DNA binding sites on the promoters of certain genes [101].

Studies have shown that H_2_S can alleviate inflammatory damage in the body through persulfidation of the STAT3/NF-κB signaling pathway. In a study by Dan et al. [102], exogenous H_2_S administration to mouse hepatocellular carcinoma cells decreased STAT3/NF-κB phosphorylation, thereby downregulating IDO1 expression and inhibiting tumor growth. In a cisplatin-induced kidney injury model, Sun et al. [103] found that STAT3/IKKβ phosphorylation and NF-κB nuclear translocation were increased compared with the control group. H_2_S inactivated IKKβ through sulfhydration at cysteine 179, thereby inhibiting NF-κB activation and the subsequent inflammatory response, thus alleviating cisplatin nephrotoxicity. In a caerulein-induced acute pancreatitis and secondary lung injury model, Mathan et al. [104] found that exogenous H_2_S reduced serum amylase, pancreatic and pulmonary myeloperoxidase (MPO) activity, and alleviated tissue edema and inflammatory cell infiltration while significantly downregulating TNF-α protein and mRNA levels. Furthermore, caerulein promoted IκBα degradation, enhanced NF-κB p65 DNA binding activity, increased STAT3 phosphorylation levels, and decreased SOCS3 protein and mRNA levels. Treatment with DAD activated PPARγ and upregulated SOCS3, thereby inhibiting STAT3 phosphorylation, while also reducing IκBα degradation and suppressing NF-κB activation. In an atherosclerosis model established by Ou et al. [105], ox-LDL treatment led to intracellular lipid droplet deposition, decreased cell viability, increased proinflammatory cytokine release, elevated OS levels, and upregulated pNF-κB p65, p JAK2, and p STAT3 protein levels. Exogenous H_2_S reversed these changes. Li et al. [106] found that exogenous H_2_S reduced reactive oxygen species (ROS) levels and STAT3/NF-κB phosphorylation in a hypoxia–reoxygenation model, suggesting that exogenous H_2_S protects aged cardiomyocytes during ischemic postconditioning, potentially through persulfidation modification.

**Figure 5:**
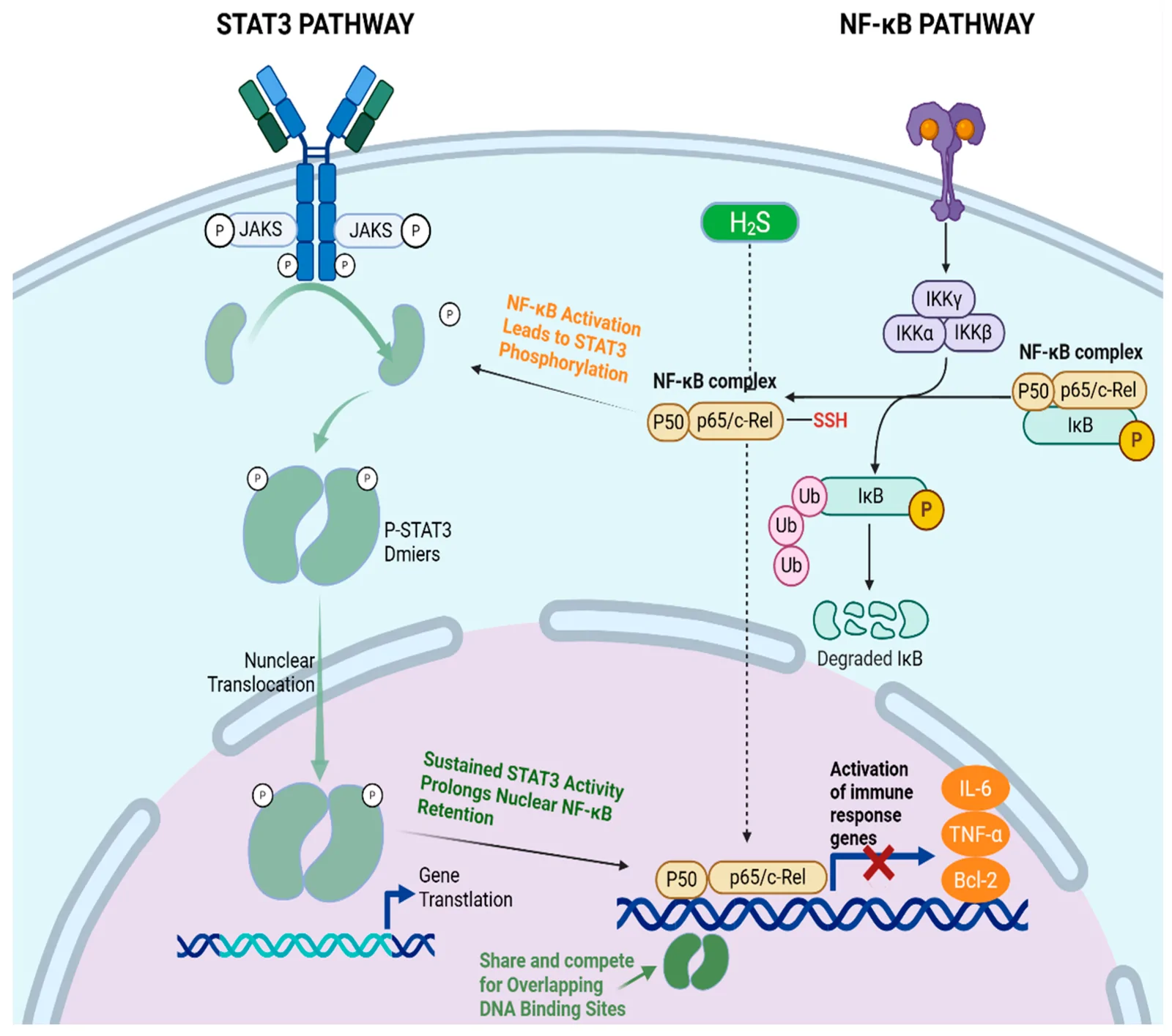
H_2_S regulates the STAT3/NF-κB signaling pathway through persulfidation modification. Abbreviations: NF-κB: nuclear factor κB; IκB: inhibitor of NF-κB; STAT: signal transducer and activator of transcription. Created in BioRender. Liang, K. (2026) https://BioRender.com/w2qn7za (accessed on 22 July 2026).

In summary, existing studies have clarified the core mechanism by which H_2_S regulates the STAT3/NF-κB signaling pathway through persulfidation modification and have validated its protective effects in various disease models, including cancer, kidney injury, pancreatitis, atherosclerosis, and myocardial ischemia, providing new ideas and targets for the treatment of related diseases. However, the molecular details of the persulfidation modification remain unclear. Only one modification site on IKKβ has been identified. The modification sites, modes, and conformational changes of core proteins such as STAT3 and NF-κB p65, as well as the upstream and downstream regulatory factors associated with this modification, have not yet been determined (Figure 5).

### 4.5. Biological Effects of H_2_S-Mediated Persulfidation of Nrf2/NF-κB

Nuclear factor erythroid 2-related factor 2 (Nrf2) is a member of the cap-n-collar (CNC) basic leucine zipper (bZIP) transcription activator family. It contains a basic leucine zipper (bZIP) structure and six functional domains (Neh1–Neh6). The Nrf2 protein comprises multiple functional regions: the Neh1 domain binds to nuclear small Maf (sMaf) proteins to form a complex that recognizes the antioxidant response element (ARE) and initiates downstream gene transcription; the Neh2 domain is primarily responsible for binding to Keap1 protein; the Neh3 domain, located at the carboxyl terminus of Nrf2, activates downstream gene transcription; the Neh4 and Neh5 domains participate in regulating Nrf2 transcriptional activity by interacting with CREB protein; and the Neh6 domain, rich in serine residues, regulates Nrf2 degradation in a Keap1 independent manner, thereby inhibiting its function. Keap1 (Kelch-like ECH-associated protein 1) is an inhibitor of Nrf2 and plays a critical role in regulating Nrf2 activity [107]. Under resting conditions, Nrf2 forms a complex with Keap1 in the cytoplasm, and Keap1 acts as an adaptor for Cullin3-based ubiquitin ligase, promoting Nrf2 ubiquitination and degradation. However, under stress conditions, cysteine residues of Keap1 are oxidized, leading to conformational changes in Keap1, which reduce Nrf2 ubiquitination levels. Nrf2 is then released from the complex and translocates to the nucleus, where it binds to the ARE in association with cofactors (e.g., sMAF) and activates downstream target genes, upregulating the expression of nicotinamide adenine dinucleotide phosphate (NADPH), NAD(P)H: quinone oxidoreductase 1 (NQO1), glutathione S-transferase (GST), heme oxygenase-1 (HO-1), and superoxide dismutase (SOD), among others [108].

Under normal conditions, Nrf2 and NF-κB coordinate and interact with each other to maintain cellular homeostasis. However, this balance is disrupted under pathological conditions. Nrf2 upregulates antioxidant enzymes and GSH levels, neutralizing excess reactive oxygen species (ROS), thereby suppressing NF-κB activation. Conversely, when Nrf2 is deficient, excess ROS cannot be neutralized, leading to increased IκBα phosphorylation and subsequent degradation, ultimately promoting the transcription of proinflammatory cytokines. In addition, Keap1 can directly bind to IKKβ and link to the CUL3 RBX1 ubiquitin ligase complex, regulating IKKβ ubiquitination and degradation, thereby activating a cascade of downstream responses [109]. Numerous studies have demonstrated that H_2_S-mediated persulfidation of the Nrf2–NF-κB axis plays an important role in antioxidative stress. For example, in a uranium poisoning model established by Wei et al. [110], NaHS treatment restored uranium-induced abnormalities in blood urea nitrogen and creatinine, decreased malondialdehyde (MDA) content, increased GSH levels and antioxidant enzyme activities, promoted Nrf2 nuclear translocation, upregulated the expression of downstream antioxidant enzymes HO-1, NQO1, GCLC, and TXNRD 1, reduced NF-κB p65 nuclear translocation, and alleviated uranium-induced renal histopathological changes. In a subsequent kidney injury model, Waz et al. [111] found that compared with the control group, the model group exhibited significantly elevated serum urea and creatinine, renal tubular degeneration and dilation, decreased p Nrf2 and HO-1 expression, and increased NF-κB expression. NaHS treatment reversed these changes and ameliorated pathological damage, suggesting that exogenous H_2_S plays a protective role against OS, with a mechanism possibly involving S-sulfhydration. Furthermore, in an alloxan-induced diabetic model [112], NaHS improved blood glucose, blood lipids, and insulin levels in rats while decreasing MDA and NO, increasing SOD, CAT, and GPx activities and Nrf2 levels, inhibiting NF-κB p65 activity, and alleviating diabetes-induced uterine pathological damage. In a human gastric epithelial cell ischemia–reperfusion (I–R) model, Guo et al. [113] found that NaHS intervention reduced I–R-induced elevations in ROS and MDA, increased GSH levels, inhibited NF-κB p65 nuclear translocation, and promoted Keap1 persulfidation, thereby facilitating Nrf2 nuclear translocation and ultimately alleviating I–R injury in gastric epithelial cells. In a study by Xie et al. [114] using streptozotocin (STZ)-induced diabetic LDLr^−^/^−^ mice, GYY4137 treatment significantly reduced aortic plaque area. However, the protective effect of H_2_S was completely absent in Nrf2-knockout (LDLr^−^/^−^Nrf2^−^/^−^) mice or cells, indicating that H_2_S requires Nrf2 to exert its protective effects. Moreover, exogenous H_2_S S-sulfhydrated Keap1 at Cys151, thereby disrupting the Keap1–Nrf2 complex, activating the Nrf2 signaling pathway, upregulating the expression of downstream antioxidant genes (GCLC, GCLM, GR), increasing GSH levels, counteracting OS, regulating cellular senescence, and ultimately slowing the progression of diabetes-accelerated atherosclerosis.

**Figure 6:**
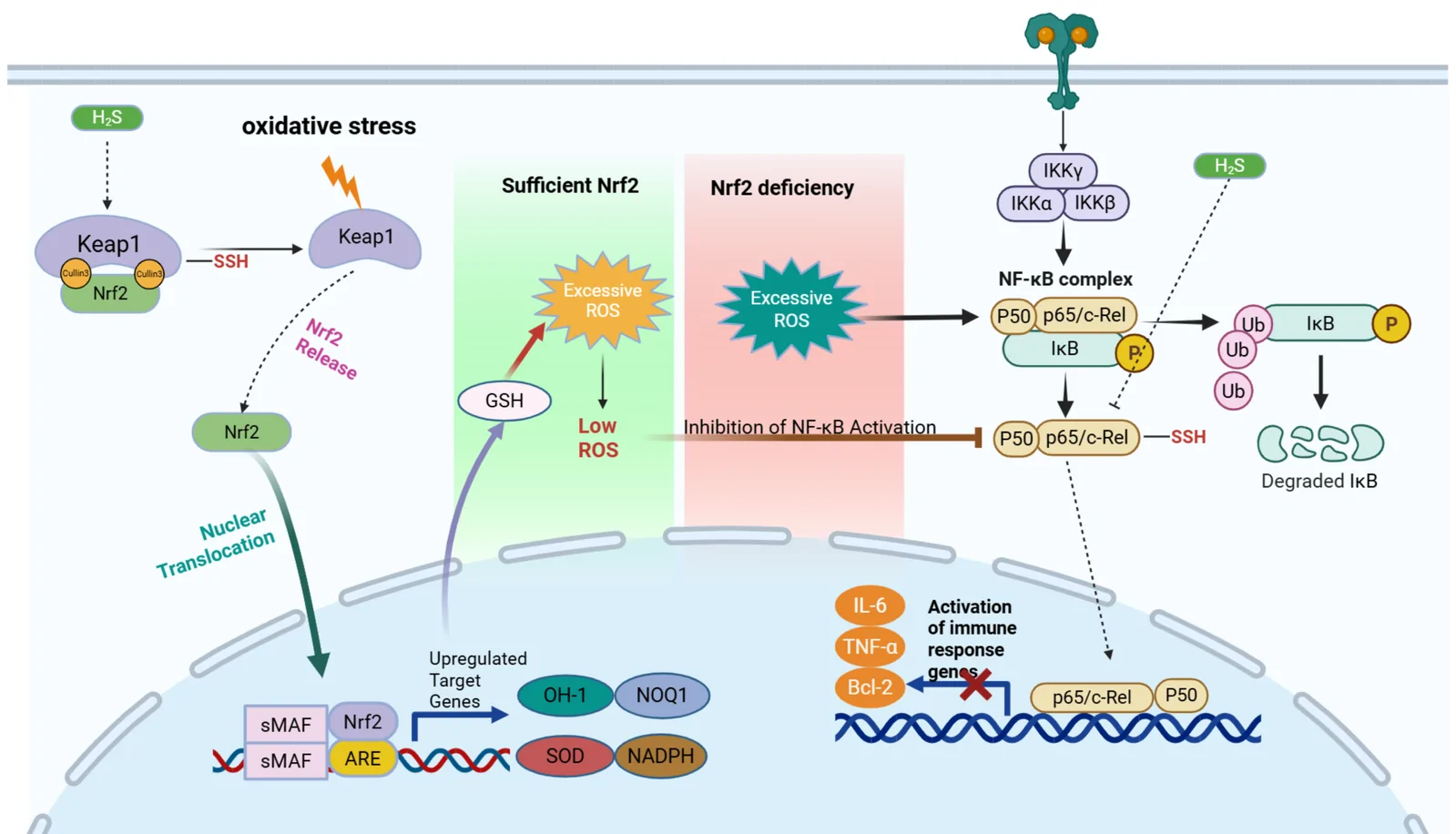
H_2_S regulates the Nrf2/NF-κB signaling pathway through persulfidation modification. Abbreviations: NF-κB: nuclear factor κB; IκB: inhibitor of NF-κB; Nrf2: nuclear factor erythroid 2-related factor 2; Keap1: Kelch-like ECH-associated protein 1. Created in BioRender. Liang, K. (2026) https://BioRender.com/w2qn7za (accessed on 22 July 2026).

In summary, H_2_S plays an important role in models of kidney injury, diabetes, atherosclerosis, and ischemia–reperfusion through persulfidation modification of the Nrf2/NF-κB signaling pathway, providing experimental evidence for the protection against these diseases (Figure 6).

### 4.6. Biological Effects of H_2_S-Mediated Persulfidation of SIRT/NF-κB

Sirtuins (SIRTs) are a family of histone deacetylases. To date, seven SIRT family proteins have been identified (SIRT1 to SIRT7), each with distinct subcellular localizations. SIRT1 and SIRT2 are found in the nucleus and cytoplasm, SIRT3, SIRT4, and SIRT5 are localized in mitochondria, and SIRT6 and SIRT7 are located in the nucleus. Furthermore, they participate in different physiological processes. SIRT1 is the most extensively studied and primarily functions in DNA repair, maintenance of cardiovascular homeostasis, anti-inflammation, and antioxidative stress. SIRT2 plays a role in lipid metabolism and is also closely associated with anti-inflammatory and antioxidative stress processes. SIRT3 mainly functions in mitochondria as a key regulator of mitochondrial metabolism, enhancing cellular energy metabolism efficiency and improving mitochondrial function. SIRT4 plays a critical role in suppressing tumor formation and promoting autophagy: it limits cancer cell proliferation and reduces the probability of tumorigenesis. SIRT5 is important in fatty acid metabolism and liver protection: it improves hepatic metabolic function and reduces fat accumulation in the liver, exerting positive effects on the prevention and alleviation of fatty liver disease. SIRT6 is primarily involved in glucose metabolism and insulin regulation: it promotes insulin release, helps regulate blood glucose levels, and prevents hyperglycemia and diabetes. SIRT6 also has anti-inflammatory and antioxidative stress effects. SIRT7 mainly functions in cardioprotection and cellular homeostasis: it protects cardiomyocytes from OS and inflammatory injury and is also closely linked to DNA repair [115,116].

SIRTs primarily inhibit NF-κB activity by deacetylating the p65 subunit of NF-κB. When cells are stimulated by external signals, IκB kinase (IKK) phosphorylates IκB, leading to IκB ubiquitination and proteasomal degradation, which releases NF-κB and allows its translocation to the nucleus to activate proinflammatory gene transcription. SIRT suppresses the DNA binding ability and transcriptional activity of nuclear p65 through deacetylation, thereby downregulating proinflammatory cytokines [117]. In recent years, numerous studies have shown that H_2_S plays an important role in anti-inflammation and antioxidative stress by S-sulfhydrating the SIRT/NF-κB signaling pathway [118]. In a diabetic model of mouse hippocampal neurons, Li et al. [119] found that compared with the control group, the model group exhibited significantly increased levels of inflammatory cytokines such as IL-1β and IL-6 and increased phosphorylation of NF-κB p65, along with decreased SIRT1 expression. NaHS treatment reversed these effects and alleviated the inflammatory response. In a subsequent study using a trimethylamine N-oxide (TMAO)-induced mouse macrophage inflammation model, Liu et al. [120] found that exogenous H_2_S inhibited TMAO-induced p65 NF-κB phosphorylation and increased persulfidation levels of SIRT1. Moreover, NaHS reduced the expression of proinflammatory cytokines such as IL-1β and IL-6 and increased the levels of anti-inflammatory cytokines such as IL-4 and IL-10. In addition, El Boghdady et al. [121] used a cuprizone-induced mouse model of multiple sclerosis and found that the cuprizone group showed decreased levels of myelin basic protein (MBP), oligodendrocyte transcription factor 2 (Olig2), and ciliary neurotrophic factor (CNTF), as well as decreased expression of autophagy proteins such as ULK1 and beclin1 and SIRT1 protein. Regarding OS, the cuprizone group exhibited decreased GSH and TAC levels and increased NF-κB expression. Treatment with diallyl disulfide (DADS) or S-adenosyl L-methionine (SAMe) enhanced autophagy protein expression, improved antioxidant capacity, inhibited inflammatory factors, and restored and improved motor coordination and myelin structure. Du et al. [122] established an atherosclerosis model and found that NaHS and GYY4137 significantly reduced plaque area in ApoE^−/−^ mice. Exogenous H_2_S S-sulfhydrated SIRT1 at Cys371/374 and Cys395/398, leading to deacetylation of the NF-κB p65 subunit, inhibition of its activity, reduced expression of inflammatory cytokines, and suppression of atherosclerosis progression. Yuan et al. [123] found that H_2_S S-sulfhydrated SIRT1, reducing the acetylation level of FOXO1, significantly increasing the expression of antioxidant enzymes such as MnSOD and CAT, and ultimately reducing intracellular ROS levels, thereby counteracting OS and delaying cellular senescence. Furthermore, in a cisplatin-induced acute kidney injury model, Yuan et al. [124] found that H_2_S S-sulfhydrated SIRT3 at Cys256, Cys259, Cys280, Cys283, and other sites, enhancing its deacetylase activity, improving mitochondrial morphology, function, and antioxidant capacity and ameliorating renal histopathological damage, thereby alleviating cisplatin-induced kidney injury.

**Figure 7:**
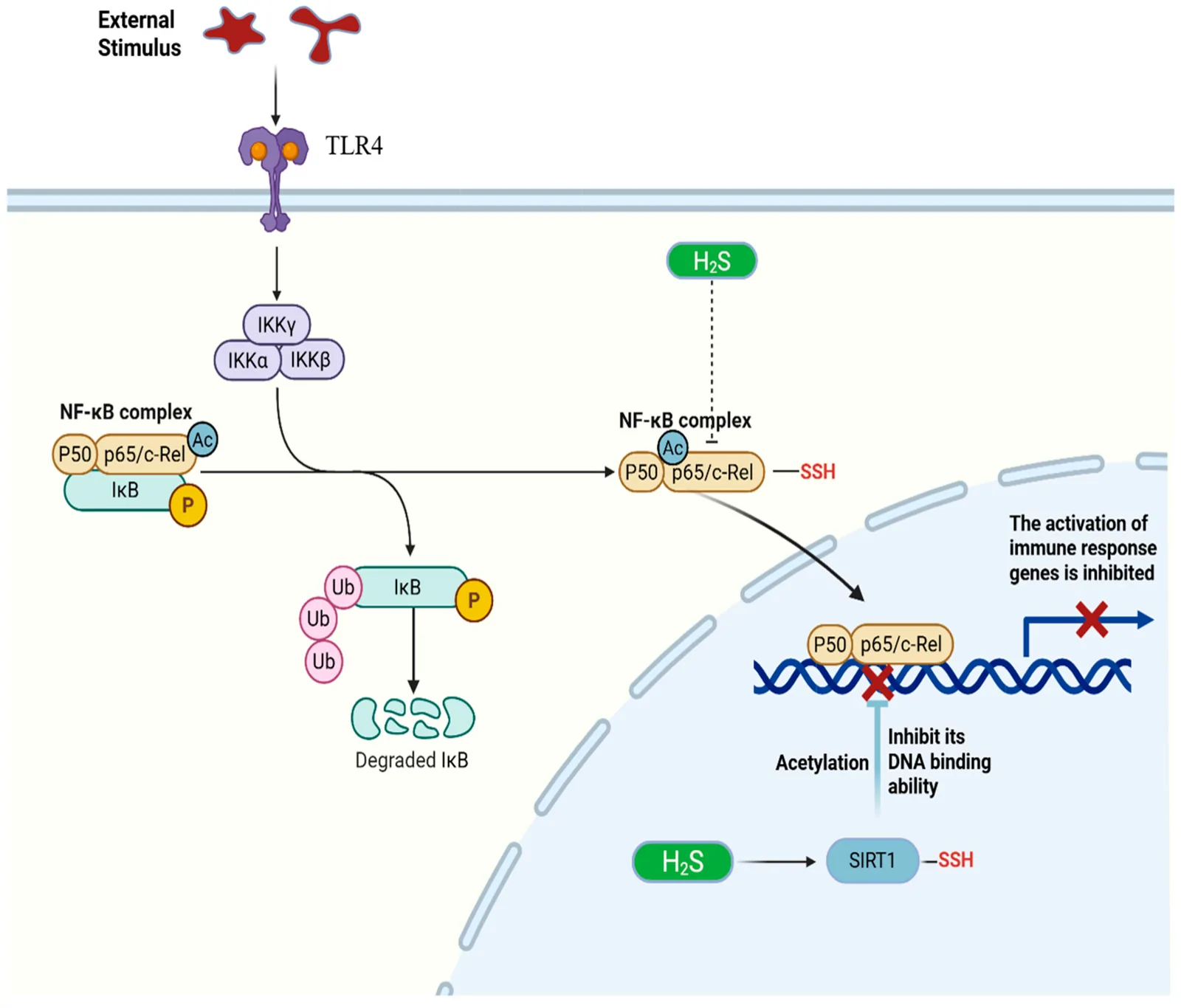
H_2_S regulates the SIRT/NF-κB signaling pathway through persulfidation modification. Abbreviations: NF-κB: nuclear factor κB; IκB: inhibitor of NF-κB; SIRT: sirtuin (silent information regulator). Created in BioRender. Liang, K. (2026) https://BioRender.com/w2qn7za (accessed on 22 July 2026).

The above studies indicate that H_2_S plays an important role in diabetes, cellular inflammatory responses, atherosclerosis, and antioxidative stress through persulfidation modification of the SIRT/NF-κB signaling pathway, providing new targets and therapeutic strategies for the treatment of diseases such as diabetes, neuroinflammation, and multiple sclerosis (Figure 7).

### 4.7. Biological Effects of H_2_S-Mediated Persulfidation of NLRP3/NF-κB

NOD-like receptor protein 3 (NLRP3) is composed of a cytoplasmic multiprotein complex and resides in the cytoplasm [125]. It contains three domains: a C-terminal leucine-rich repeat (LRR) domain, an N-terminal pyrin domain (PYD), and a central nucleotide binding and oligomerization domain (NACHT) [126]. The activation of NLRP3 occurs primarily through canonical and non-canonical pathways. The canonical pathway is initiated by pathogen-associated molecular patterns (PAMPs), damage-associated molecular patterns (DAMPs), or inflammatory factors, which activate NF-κB signaling by binding to cell surface receptors, upregulating NLRP3 and other proteins to complete the priming step. Subsequently, signals such as potassium efflux, mitochondrial reactive oxygen species (mtROS) released from damaged mitochondria, and cathepsin B released from ruptured lysosomes activate NLRP3, leading to the assembly of a complex with apoptosis-associated speck-like protein containing a CARD (ASC) and pro-caspase 1, ultimately activating caspase 1 and triggering pyroptosis [127]. The non-canonical pathway is primarily mediated by lipopolysaccharide (LPS). LPS enters the cytoplasm directly and binds to and activates pro-caspase 11 (or caspase 4/5 in humans), thereby inducing pyroptosis [128].

Wang et al. [129] established a palmitic acid (PA)-induced inflammatory model in human hepatocellular carcinoma cells and found that NaHS treatment significantly inhibited PA-induced inflammatory cytokine expression and release and dose-dependently suppressed NLRP3 inflammasome expression, NF-κB pathway activation, and p65 nuclear translocation, thereby alleviating hepatocyte inflammation. In an LPS-induced mouse depression model, Bao et al. [130] found that exogenous H_2_S inhibited LPS-induced NF-κB p65 phosphorylation in the hippocampus, as well as the protein expression of NLRP3, ASC, and caspase 1. It also reduced LPS-induced GSDMD N protein expression and the number of TUNEL positive cells in the hippocampal CA1, CA3, and DG regions. Furthermore, NaHS ameliorated LPS-induced mitochondrial swelling, blurred cristae structure, and improved membrane potential and ATP production. These findings suggest that exogenous H_2_S prevents LPS-induced depression-like behavior by inhibiting activation of the hippocampal NF-κB/NLRP3 inflammasome pathway, reducing pyroptosis, and improving mitochondrial morphology and function, with the mechanism potentially involve over-vulcanization modification. Wu [131] established an LPS+ATP model and found that NaHS pretreatment significantly reduced LPS+ATP-induced expression of NF-κB and NLRP3 inflammasome related proteins. In addition, exogenous H_2_S reversed LPS+ATP-induced autophagy inhibition, thereby alleviating the inflammatory response. Huang et al. [132] used rat embryonic cardiomyocyte cell line H9c2 to establish a high-glucose-induced cardiotoxicity model and found that the high-glucose group exhibited decreased cell viability, increased apoptosis, elevated ROS levels, and decreased mitochondrial membrane potential. Moreover, NF-κB, NLRP3 inflammasome, and downstream inflammatory factors were upregulated. NaHS pretreatment reversed these effects, thereby alleviating high-glucose-induced cardiomyocyte inflammation and apoptosis. Furthermore, Li et al. [81] established a sepsis model and found that exogenous H_2_S significantly inhibited the expression of pNF-κB, NLRP3, and downstream inflammatory factors while markedly improving mouse survival and alleviating histopathological conditions such as pulmonary edema and vascular leakage. In further cellular experiments, GYY4137 similarly inhibited NLRP3 inflammasome release and reduced the cellular inflammatory response. In an LPS/ATP-induced macrophage pyroptosis model, Ma et al. [133] found that GYY4137 reduced the expression of NLRP3, ASC, and GSDMD, restored LPS/ATP-induced mitochondrial structural damage, decreased membrane potential, and reduced respiratory chain complex activity. In a subsequent mouse model of osteoarthritis, GYY4137 treatment alleviated pathological damage and cartilage degradation, inhibited caspase 1 expression in the synovium, and reduced p65 phosphorylation and its DNA binding activity through persulfidation at Cys38 of p65, ultimately inhibiting synovial macrophage pyroptosis, alleviating cartilage degradation, and slowing osteoarthritis (OA) progression.

**Figure 8:**
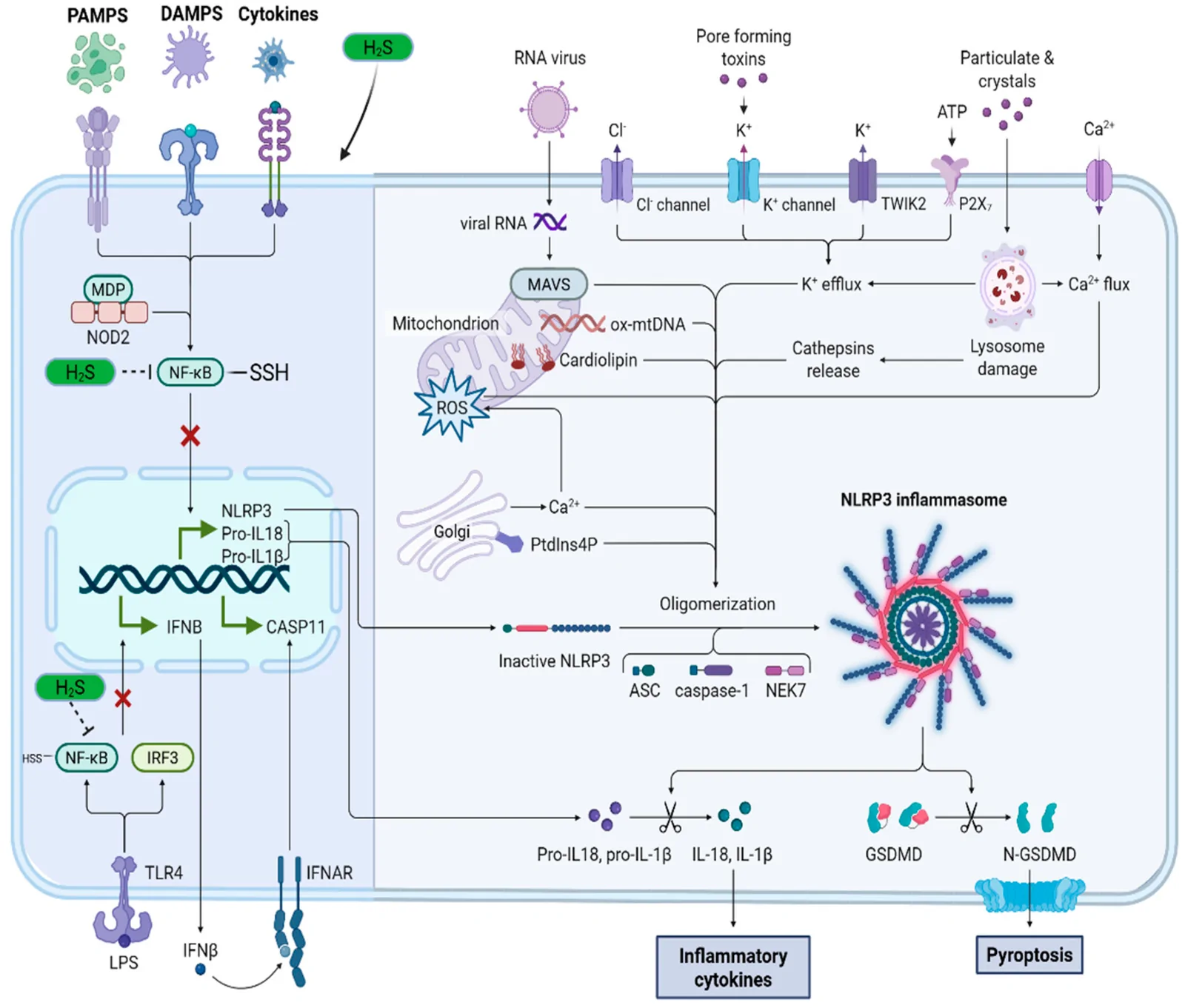
H_2_S regulates the NLRP3/NF-κB signaling pathway through persulfidation modification. Abbreviations: NF-κB: nuclear factor κB; IκB: inhibitor of NF-κB; NLRP3: NOD-like receptor protein 3. Created in BioRender. Liang, K. (2026) https://BioRender.com/w2qn7za (accessed on 22 July 2026).

To date, it has been demonstrated in various pathological models, including liver inflammation, neuropsychiatric injury, myocardial glycolipid toxicity injury, sepsis, and osteoarthritis, that H_2_S, using persulfidation as the core regulatory mechanism, targets and modifies key sites of NF-κB, inhibits NLRP3 inflammasome assembly and activation at the transcriptional level and simultaneously modulates mitochondrial function, autophagy, and pyroptosis, thereby achieving multi-target anti-inflammatory organ protection (Figure 8).

### 4.8. Biological Effects of H_2_S-Mediated Persulfidation of TNF-α/NF-κB

TNF-α is a cytokine of the TNF superfamily, composed of 157 amino acids and featuring a unique trimeric structure. TNF-α exerts its effects by binding to two distinct receptors: type I tumor necrosis factor receptor (TNFR1) and type II tumor necrosis factor receptor (TNFR2). TNFR1 recruits various adaptor proteins, including TNFR-associated death domain (TRADD), TNFR-associated factor 2 or 5 (TRAF2/5), receptor-interacting protein (RIP) kinase, and inhibitors of apoptosis proteins (IAPs), to form a complex. This complex subsequently activates IKK, which then activates the NF-κB pathway by degrading the IκB complex, thereby releasing the p50 subunit. p50 translocates to the nucleus and directly regulates gene expression. TNFR2, unlike TNFR1, lacks a death domain and therefore cannot regulate apoptosis. However, activated TNFR2 recruits the adaptor proteins TRAF2 and IAPs and can also activate NF-κB signaling via the canonical pathway. In addition, TNFR2, TRAF2, and IAPs can form a complex with NIK. Upon stimulation, NIK is released from the complex and activated. Active NIK induces the non-canonical NF-κB pathway through IKKα, ultimately generating the transcriptionally active p52 subunit and inducing gene transcription [134,135]. It is noteworthy that NF-κB activation requires stimulation by TNF-α, and activated NF-κB promotes the transcription of proinflammatory cytokines such as TNF-α, which in turn restimulates the NF-κB pathway. This creates a vicious cycle that continuously amplifies the inflammatory cascade. Numerous studies have indicated that H_2_S alleviates the body’s inflammatory response through S-sulfhydration modification [136].

**Figure 9:**
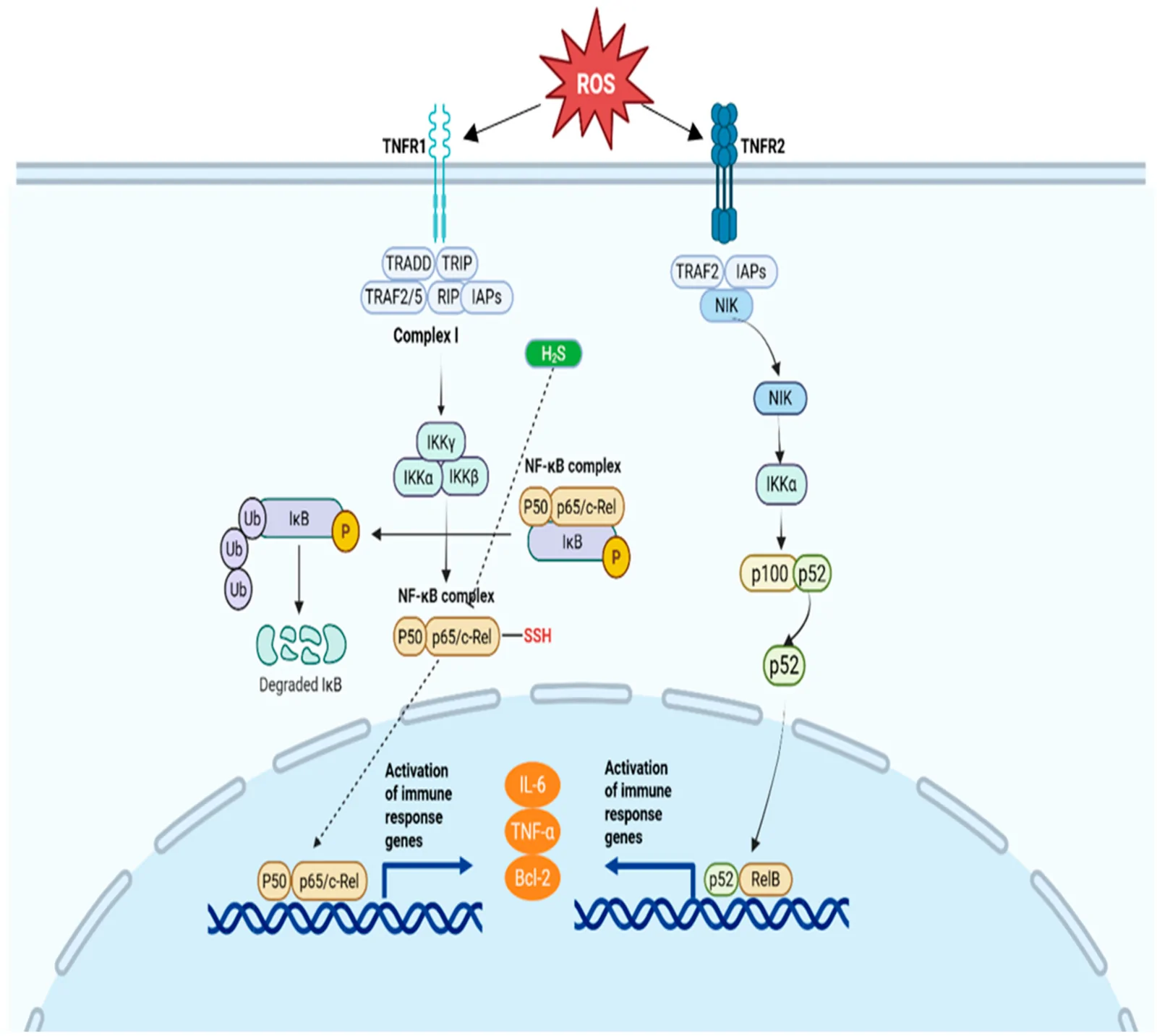
H_2_S regulates the TNF-α/NF-κB signaling pathway through persulfidation modification. Abbreviations: NF-κB: nuclear factor κB; IκB: inhibitor of NF-κB; TNFR1: type I tumor necrosis factor receptor; TNFR2: type II tumor necrosis factor receptor. Created in BioRender. Liang, K. (2026) https://BioRender.com/w2qn7za (accessed on 22 July 2026).

Du et al. [137] established a macrophage atherosclerosis model and found that oxidized low-density lipoprotein (ox-LDL) increased the levels of monocyte chemoattractant protein 1 (MCP-1), TNF-α, and migration inhibition factor (MIF), whereas NaHS reversed this effect. Exogenous H_2_S S-sulfhydrated the p65 subunit at Cys38, inhibiting its phosphorylation and IκBα activation, thereby suppressing NF-κB activity and alleviating inflammatory damage. Chen et al. [138] established a urogenital sepsis model in male rabbits and found that TNF-α and NF-κB expression in renal tissue were elevated in the sepsis group. NaHS treatment reduced TNF-α and NF-κB expression and also alleviated renal histopathological damage, with the mechanism potentially involving over-vulcanization modification. In an irritable bowel syndrome model, Yang et al. [139] found that the infection group exhibited increased levels of inflammatory cytokines including TNF-α, elevated MDA, and decreased T-AOC and GSH-Px activities. Infection also activated NF-κB and reduced IκBα/IκBβ levels. NaHS treatment significantly alleviated inflammatory infiltration, reduced the expression of certain inflammatory cytokines and TNF-α, inhibited NF-κB activity, and upregulated IκBα, ultimately attenuating the inflammatory response and oxidative damage. Liu et al. [140] established an acute myocardial ischemia model and found that exogenous H_2_S ameliorated the pathological damage induced by myocardial ischemia, including disordered myocardial fiber arrangement, mitochondrial swelling, and cristae loss while also reducing serum levels of TNF-α and other inflammatory cytokines. Furthermore, NF-κB expression was significantly increased in the ischemia group and NaHS treatment reduced its expression, suggesting that S-sulfhydration modification may play an important role. Zhang et al. [141] used a monocrotaline-induced rat pulmonary hypertension model and found that H_2_S S-sulfhydrated IKKβ at Cys179 and p65 at Cys38, directly inhibiting IKKβ activity and blocking the NF-κB signaling pathway, thereby reducing intracellular inflammatory cytokine and TNF-α levels. However, when the Cys179 site was mutated, the above changes did not occur. Thus, H_2_S can alleviate pulmonary artery endothelial cell inflammation, vascular remodeling, and pulmonary hypertension by S-sulfhydrating the TNF-α/NF-κB signaling pathway (Figure 9).

### 4.9. Biological Effects of H_2_S-Mediated Persulfidation of IL/NF-κB

Interleukins (ILs) are a family of cytokines secreted by immune cells and play essential roles in inflammatory responses, immune cell differentiation and activation, and the overall homeostasis of the immune system. To date, at least 38 interleukins have been identified and classified into six major families based on structure and function: IL-1, IL-2, IL-6, IL-10, IL-12, and IL-17 [142]. The IL-1 family is primarily derived from mononuclear phagocytes, as well as lymphocytes, endothelial cells, and keratinocytes. At low concentrations, IL-1 mainly exerts immunomodulatory effects, whereas at high concentrations it induces inflammatory responses [143]. The IL-2 family is mainly produced by CD4^+^ T cells and plays an immunomodulatory role, suppressing immune responses at low concentrations and promoting them at high concentrations [144]. The IL-6 family is produced by monocytes/macrophages, endothelial cells, adipocytes, and hematopoietic cells, and plays critical roles in inflammation regulation, immune responses, and hematopoiesis. It transmits downstream signals through three pathways: classical signaling, trans signaling, and trans presentation signaling [145]. The IL-10 family is produced by Th2 cells, mononuclear macrophages, B cells, and epithelial cells. It exerts anti-inflammatory effects by inhibiting the secretion of proinflammatory cytokines from monocytes and macrophages and also modulates immune responses by suppressing Th1 and Th17 cell responses while promoting Th2 responses [146]. The IL-12 family is produced by dendritic cells, macrophages, neutrophils, and human B lymphoblasts and plays important roles in regulating innate and adaptive immune responses [147]. The IL-17 family is secreted by CD4^+^ T cells and primarily functions to activate tissue responses and guide neutrophil-mediated immune defense [148].

ILs primarily activate the NF-κB signaling pathway through the canonical pathway. For example, IL-1β binds to the IL-1 receptor (IL-1R) and forms a complex with IRKA1 and IRKA4 by recruiting the death domain of the adaptor protein MyD88. IRKA4 phosphorylates IRKA1, which then dissociates from the complex and binds to TRAF6, thereby activating and synthesizing K63 ubiquitin chains. TAK1/TAB1/TAB2 use these ubiquitin chains as a scaffold to activate IKK, ultimately leading to the release of the p50/p65 dimer, which translocates into the nucleus and initiates gene transcription [149].

**Figure 10:**
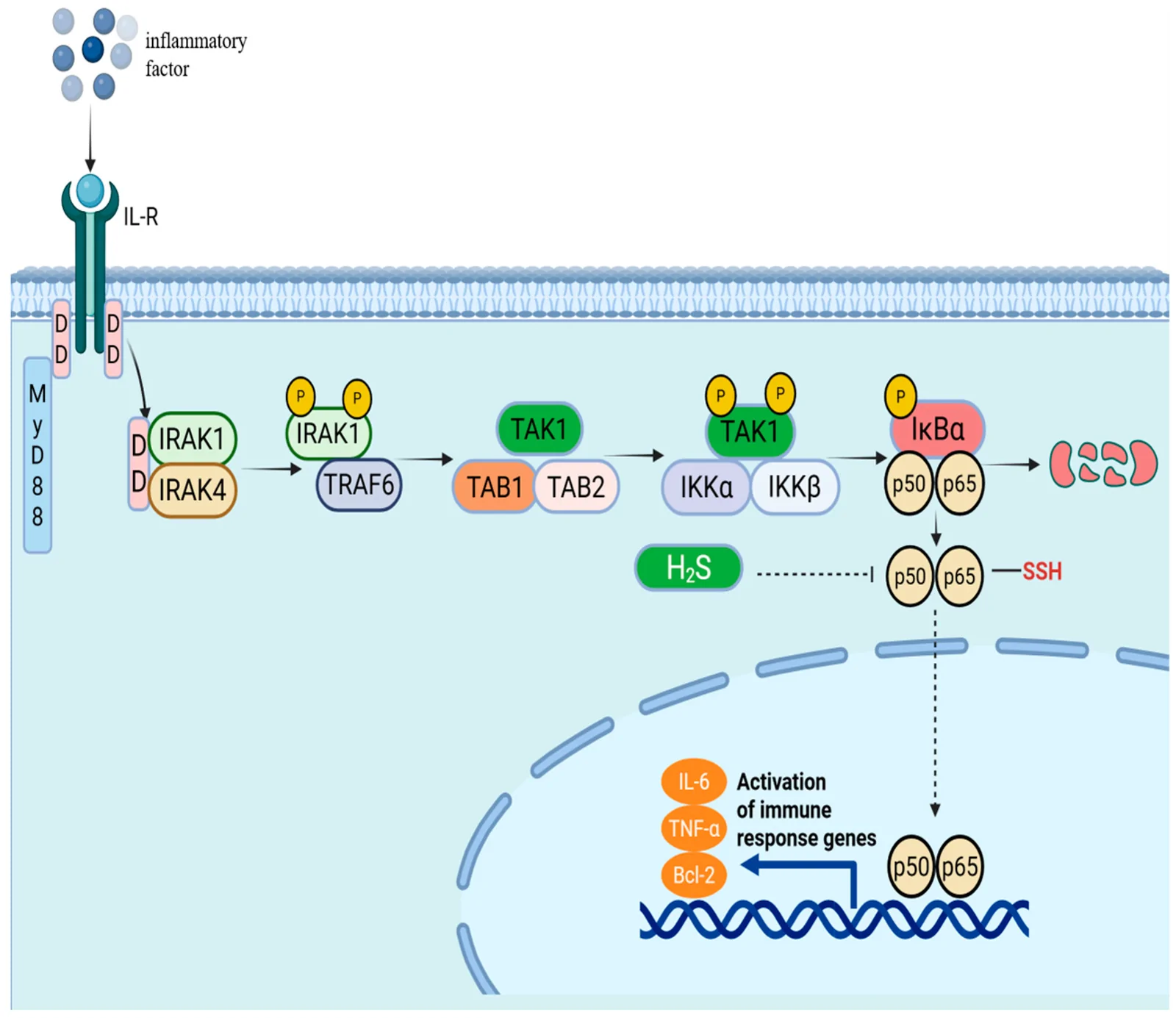
H_2_S regulates the IL/NF-κB signaling pathway through persulfidation modification. Abbreviations: NF-κB: nuclear factor κB; IκB: inhibitor of NF-κB; IL: interleukin. Created in BioRender. Liang, K. (2026) https://BioRender.com/w2qn7za (accessed on 22 July 2026).

Xu et al. [150] established a high-glucose-induced injury model and found that glucose treatment significantly increased p-NF-κB p65 levels as well as IL-1β and IL-6 levels. NaHS pretreatment reversed this effect, increased cell viability, reduced the number of apoptotic cells and cleaved caspase 3 expression. In addition, exogenous H_2_S reduced ROS production and maintained mitochondrial membrane potential, thereby alleviating high-glucose-induced cardiac injury. In an aortic valve calcification model established by Éva and colleagues [151], IL-1β levels in calcified tissues were significantly higher than those in the control group, and NF-κB protein expression was also increased. H_2_S inhibited the activation of the NF-κB signaling pathway, preventing the nuclear translocation of the osteogenic transcription factor Runx2, thereby blocking the transformation of aortic valve interstitial cells into osteoblast-like cells and inhibiting valve calcification. This process is speculated to involve persulfidation modification. In a study by Xu et al. [152], doxorubicin (DOX) upregulated NF-κB p65 expression in cardiomyocytes, significantly increased the secretion of IL-1β and IL-6, increased the number of apoptotic cells, and decreased cell viability. NaHS treatment suppressed the upregulation of p-p65 expression, alleviated DOX-induced inflammation and cell damage, reduced the secretion levels of IL-1β and IL-6, decreased the number of apoptotic cells, and increased cell survival. Zhong et al. [153] used a streptozotocin-induced type 1 diabetes model and found that, compared with the control group, the model group exhibited elevated serum levels of IL-1β, IL-8, and dsDNA, as well as increased expression of CXCR2, p-IκBα, and p-NF-κB. NaHS treatment significantly reduced these levels. In further in vitro experiments, glucose induced upregulation of Bax, IL-1β, and IL-8 expression and downregulation of Bcl-2 in endothelial cells, and exogenous H_2_S treatment reversed these changes. Furthermore, in a sepsis model established by cecal ligation and puncture, Xu et al. [154] found that NaHS increased IL-10 levels and inhibited NF-κB expression, while also reducing pathological damage. These findings suggest that exogenous H_2_S protects against sepsis-induced myocardial injury by inhibiting NF-κB expression and upregulating the anti-inflammatory cytokine IL-10, with the mechanism speculated to involve H_2_S-mediated persulfidation modification (Figure 10).

In summary, H_2_S-mediated persulfidation can regulate the NF-κB signaling pathway through multiple pathways, including STAT3, NLRP3, Nrf2, SIRT, MAPK, PI3K/Akt, TLR4, TNF-α, and IL, thereby achieving various biological effects such as anti-inflammatory, anti-apoptotic, and anti-oxidative stress responses, ultimately exerting protective effects (Table 3).

**Table 3:** Biological effects of H_2_S mediated by persulfidation modification.

Signaling Pathway	Subject	Model	H_2_S Donor	Administration Method	Mechanism of Action	Reference
TLR4/NF-κB	MAC-T cells	Inflammatory model	NaHS	Cells were pre-incubated with NaHS for 2 h, followed by co-treatment with LPS.	ROS, p-p65, TLR4↓	[68]
	SH–SY5Y cells	Parkinson’s model	NaHS	Directly added to the medium	TLR4, iNOS, p-IκB, p-p65 ↓	[69]
	pregnant C57BL/6 mice; HUSMCs cells	Infectious preterm birth model	NaHS	Subcutaneous injection; directly added to the medium	ASC, caspase 1, T LR4, p-p65 ↓	[70]
	Dahl salt-sensitive rats	Hypertension model	GYY4137	Dissolved in artificial cerebrospinal fluid (aCSF) and administered via an osmotic pump	p-p65, p-IKKβ, TLR4, MyD88 ↓	[71]
	SD rats	COPD model	H_2_S	Inhalation	GSDMD-N, cleaved caspase 1, LDH, TLR4, p-p65 ↓	[72]
	Wistar male rats	Hepatotoxicity model	NaHS	IP	TBARS, Nox, TLR2, TLR4, p-JNK, p-p65 ↓; GSH ↑	[73]
	KM mice	Alcoholic liver disease model	NaHS	IP	AST, ALT, MDA, TLR4, NF-κB ↓; GSH, CSE ↑	[74]
PI3K/Akt/NF-κB	SD rats	Sepsis model	NaHS	IP	p-PI3, p-Akt, Bcl-2 ↑; NF-κB, Bax, caspase 3 ↓	[80]
	C57BL/6J mice; RAW 264.7 cells	ALI model	GYY4137	IP; directly added to the medium	p-PDGFRβ, p-Akt, p-p65 ↓	[81]
	MAC-T cells	Inflammatory model	NaHS	Directly added to the medium	p-Akt, NF-κB ↓	[82]
	Male white big-eared rabbits	Portal hypertension model	NaHS	IP	p-AKT, NF-κB ↑; IκBα, Bcl-2 ↓	[83]
	MC3T3-E1 cells; hFOB 1.19 cells	Osteoblast injury model	NaHS or Na_2_S	NaHS was added 1 h prior to LPS treatment.	p-Akt, p-IκB ↓	[84]
	Rat pancreatic acinar cells	Acute pancreatitis model	NaHS or Na_2_S	Directly added to the medium	p-Akt, p-p65 ↓	[85]
	SD rats	Severe acute pancreatitis model	NaHS	IP	MPO, p-Akt, NF-κB ↓; IκBα ↑	[86]
	C57BL/6J mice	Myocardial injury model	NaHS	IP	MDA, Bax/Bcl-2, cleaved caspase 3, PI3K/AKT, p-p65 ↓; SOD ↑	[87]
	HL-1 cells	I–R injury	NaHS	NaHS was added 30 min before hypoxia treatment.	cleaved caspase 3, p-p65 ↓, Bcl-2, PI3K/Akt ↑	[88]
MAPK/NF-κB	NRK-52E; Wistar rats	Nephrotoxicity model; chronic renal failure model	NaHS	Co-treatment with NaHS was performed for 24 h during gentamicin-induced injury. IP	Bax, caspase 3, cleaved caspase 3, ROS, MDA, p-ERK, p-JNK, p-p38, p65 ↓; SOD, GSH-Px ↑	[94]
	L6 cells; Wistar rats	Inflammatory model; diaphragmatic dysfunction model	NaHS	NaHS was co-treated for 24 h during LPS stimulation. IP	Bax, caspase 3, cleaved caspase 3, p-ERK, p-JNK, p-p38, ROS, p-IKKα/β, p-IκBα, p-p65 ↓ SOD, GSH-Px ↑	[95]
	Broilers	H_2_S gas exposure model	H_2_S	Direct inhalation	GSH, CAT, T-AOC, SOD, IκBα ↓; iNOS, NO, H_2_O_2_, MDA, p-IKKα/β, p-p65, p-ERK, p-JNK, p-p38 ↑	[96]
	C57BL/6J mice	Diabetes model	NaHS	IP	p-p38MAPK, p-p65 ↓	[97]
	NIH3T3 cells	Cellular fibrosis model	NaHS	Cells were pretreated with NaHS for 1 h, followed by treatment with TGF β1 for 24 h.	p-p38MAPK, p-p65 ↓	[98]
STAT3/NF-κB	C57BL/6J mice; MCF-7; SGC-7901	HCC model	NaHS; GYY4137	IP; directly added to the medium	IDO1, p-STAT3, p-NF-κB ↓	[102]
	LLC-PK1; C57BL/6J mice	Nephrotoxicity model	NaHS; GYY4137	Pretreatment with NaHS or GYY4137 was performed for 30 min before cisplatin addition.; IP	COX-2, p-STAT3, p-IKKβ, NF-κB ↓	[103]
	Swiss albino mouse	Acute pancreatitis model	DADS	IP	p-STAT3, p-p65 ↓; PPARγ ↑	[104]
	HUVECs	Atherosclerosis model	ADT-OH	Directly added to the medium	p-STAT3, p-p65 ↓; SOD ↑	[105]
	Wistar rat cardiomyocytes	I–R model	NaHS	NaHS was added during reoxygenation.	MDA, ROS, p-p65, p-STAT3 ↓; SOD, CAT, GSH-Px↑	[106]
Nrf2/NF-κB	SD rats	Acute nephrotoxicity model	NsHS	IP	p-p65, p-IκB, Nrf2, MDA ↓, SOD, CAT, GPx, GST ↑	[110]
	Wistar rats	Acute nephrotoxicity model	NaHS	IP	NF-κB ↓; p-Nrf2, OH-1 ↑	[111]
	Wistar albino rats	Type I diabetes model	NaHS	IP	MDA, NO, p-p65 ↓; SOD, GPx, CAT, Nrf2 ↑	[112]
	GES-1 cells	I–R model	NaHS	Pretreatment with NaHS was performed for 30 min before ischemia.	ROS, MDA, p-p65, Nrf2 ↓; GSH ↑	[113]
	C57BL/6J mice; HUVECs; EA.hy926 cells	Diabetes model	GYY4137	IP; co-treatment with GYY4137 was performed for 24 h during HG+ox-LDL-induced injury.	VCAM-1, ICAM-1, p-p65, Nrf2 ↓	[114]
SIRT/NF-κB	HT-22 cells	Diabetes model	NaHS	Before high-glucose treatment, cells were pretreated with NaHS for 30 min.	p-p65 ↓; SIRT ↑	[119]
	RAW264.7 cells	Inflammatory model	NaHS	Directly added to the medium	p-p65 ↓; SIRT1 ↑	[120]
	C57BL/6 mice	Acute demyelination model	DADS	i.g.	NF-κB ↓; MBP, Olig2, CNTF, ULK1, beclin1, SIRT1, GSH, TAC ↑	[121]
	ApoE^−/−^ mice; HAECs cells; HepG2; HEK-293 cells; primary mouse peritoneal macrophages	Atherosclerosis model	NaHS; GYY4137	IP; co-treatment with NaHS was performed at the same time as cisplatin treatment.	NF-κB ↓; SIRT1 ↑	[122]
	HUVECs	Cellular senescence model	NaHS	NaHS was co-treated with H_2_O_2_.	p-p65 ↓; MnSOD, CAT, FOXO1, SIRT1 ↑	[123]
	C57BL/6 mice; HK2 cells	Acute kidney injury (AKI) model	NaHS	IP; directly added to the medium	SIRT ↑; ROS ↓	[124]
NLRP3/NF-κB	HepG2 cells	Inflammatory mode	NaHS	Before PA addition, cells were pretreated with various concentrations of NaHS for 2 h.	NLRP3, p-IKKα/β, p-p65 ↓	[129]
	ICR mice	Depression model	NaHS	IP	NLRP3, p-p65, ROS ↓; MMP, ATP ↑	[130]
	L02 cells	Inflammatory mode	NaHS	Cells were pretreated with NaHS for 30 min prior to LPS treatment.	NLRP3, NF-κB ↓	[131]
	H9c2 cells	Cardiotoxicity model	NaHS	Before high-glucose treatment, cells were pretreated with NaHS for 30 min.	NLRP3, p-p65, ROS ↓; MMP ↑	[132]
	C57BL/6J mice	Acute lung injury (ALI) model	GYY4137	IP	NLRP3, p-p65 ↓	[81]
	RAW 264.7 cells; primary mouse chondrocytes	Macrophage pyroptosis model	GYY4137	Before stimulation with LPS/ATP, cells were pretreated with GYY4137.	MMP-13, ROS, MDA, p-IκBα, p-p65 ↓; SOD ↑	[133]
TNF-α/NF-κB	THP-1 cells; RAW 264.7 cells	Inflammatory mode	NaHS	Before ox-LDL stimulation, cells were pretreated with NaHS for 30 min.	MCP-1, TNF-α, MIF, p-p65 ↓	[137]
	Rabbit	Urosepsis model	NaHS	injection into the marginal ear vein	TNF-α, NF-κB ↓	[138]
	KM mice; Caco-2 cells	IBS model; Inflammatory mode	NaHS	IP; directly added to the medium	MDA, p-p65, TNF-α↓; IκBα, SOD1, Gpx1, Gpx4 ↑	[139]
	SD rats	Myocardial ischemia model	NaHS	IP	TNF-α, NF-κB ↓	[140]
	Human PAECs cells; Wistar rats	PAH model	NaHS	Directly added to the medium; IP	p-IKKβ, pIκBα, TNF-α ↓	[141]
IL/NF-κB	H9c2 cells	High-glucose injury model	NaHS	Cells were pretreated with NaHS prior to high-glucose (HG) exposure	p-p65, ROS, MMP, IL-1β, IL-6 ↓	[150]
	AVICs cells; C57BL/6J mice	Aortic valve calcification model	AP27	Directly added to the medium; IP	p-p65, IL-1β ↓	[151]
	H9c2 cells	Inflammatory mode	NaHS	Pretreatment with NaHS for 30 min.	p-p65, IL-1β, IL-6 ↓	[152]
	Wistar rats; RAECs	Type 1 diabetes model	NaHS	IP; directly added to the medium	IL-1β, IL-8, dsDNA, CXCR2, p-IκBα, p-NF-κB, ROS ↓	[153]
	SD rats	Sepsis model	NaHS	IP	NF-κB ↓; IL-10 ↑	[154]

Note: ↑ represents increase, ↓ represents decrease.

## 5. Conclusions and Outlook

As a key gaseous signaling molecule in the body, H_2_S widely participates in the functional regulation of the NF-κB signaling pathway through persulfidation, an important post-translational protein modification. It plays an irreplaceable role in maintaining cellular homeostasis, suppressing excessive inflammation, alleviating OS, ameliorating apoptosis and pyroptosis, and protecting organ structure and function. Existing studies have systematically demonstrated that H_2_S can S-sulfhydrate specific cysteine residues on core proteins such as IKKβ, p65, Keap1, and SIRT1/3, thereby inhibiting IKK complex activation, reducing IκBα degradation, and blocking p65 phosphorylation and nuclear translocation. This upstream inhibition of NF-κB pathway overactivation downregulates the expression of proinflammatory cytokines such as TNF-α, IL-1β, IL-6, and MCP-1 and halts the inflammatory cascade. Furthermore, through cross-regulation of multiple signaling pathways, including STAT3, NLRP3, Nrf2, SIRT, MAPK, PI3K/Akt, TLR4, TNF-α, and IL-related cascades, H_2_S exerts pleiotropic protective effects in various disease models, such as atherosclerosis, sepsis, acute kidney injury, acute myocardial ischemia, acute pancreatitis, pulmonary hypertension, osteoarthritis, neuroinflammation, diabetic complications, OS, and ischemia–reperfusion injury. These effects include anti-inflammation, antioxidation, antiapoptosis, inhibition of pyroptosis, improvement of mitochondrial function, and attenuation of histopathological damage. Key modification sites identified to date include p65 Cys38, IKKβ Cys179, Keap1 Cys151, and multiple sites on SIRT1/3, providing a stable and reliable experimental basis for understanding the molecular mechanisms by which H_2_S regulates inflammation.

Despite significant progress in this field, several limitations remain that constrain the depth of mechanistic understanding and the pace of clinical translation. First, the molecular mechanisms have not been systematically or thoroughly elucidated. Most studies remain at the level of phenotypic observation of signaling pathways, and further work is needed to precisely determine the modification sites of persulfidation on key proteins, modification efficiency, conformational changes, alterations in binding affinity, and the details of upstream and downstream cascade transmission. Second, research on the physiological regulation of endogenous H_2_S is lacking. Existing findings rely largely on interventions with exogenous H_2_S donors, whereas how endogenous H_2_S synthesizing enzymes such as CBS, CSE, and 3 MST dynamically regulate the NF-κB pathway and maintain immune homeostasis under physiological and pathological conditions remains unclear. Third, the mechanisms underlying the bidirectional regulatory effects of H_2_S are unknown. H_2_S exhibits either activating or inhibitory effects on NF-κB and its related pathways depending on the model, concentration, and disease stage. The molecular switches, microenvironment-dependent mechanisms, and dose–effect relationships underlying this dual regulation have not been elucidated. Fourth, studies on cross talk among post-translational modifications are insufficient. How persulfidation antagonizes or coordinates with phosphorylation, acetylation, ubiquitination, and S nitrosylation to regulate NF-κB activity lacks systematic validation. Fifth, the foundation for clinical translation is weak. Most studies are based on cell and animal models, lacking validation using clinical tissue samples. The administration route, dosage range, intervention timing, safety, targeting, and pharmacokinetic properties of H_2_S donors have not been standardized. Sixth, the limitations of current detection techniques are evident. Existing methods for detecting S-sulfhydration suffer from insufficient specificity, high false-positive rates, inability to perform real-time in situ dynamic monitoring, and difficulty in achieving high-throughput mass spectrometry-based screening, which impairs the accuracy of mechanistic studies.

In summary, regulation of the NF-κB signaling pathway by H_2_S through persulfidation modification represents a critical node connecting gaseous signaling molecules, post-translational protein modifications, and inflammatory regulation. As research continues to advance, this axis is expected to provide novel molecular targets and therapeutic strategies for inflammation related diseases, cardiovascular diseases, metabolic disorders, neurodegenerative diseases, and organ injury, holding significant scientific value and broad prospects for clinical translation. It is worth noting that the biological effects of H_2_S exhibit a strict biphasic nature: physiological concentrations of H_2_S exert cytoprotective effects, whereas excessively high concentrations may induce reductive stress, leading to metabolic reprogramming and cellular dysfunction. Future studies are needed to further define the concentration thresholds, molecular mechanisms, and pathophysiological significance of H_2_S-mediated reductive stress.

## Figures and Tables

**Table 1:** Methods for the quantitative detection of H_2_S.

Method Category	Detailed Method	Operating Steps	Applicable Samples	Reference
Direct ultraviolet-visible spectrophotometric measurement	Direct ultraviolet-visible spectrophotometric measurement	Observe the absorbance of sulfide species in the ultraviolet region (214–300 nm) of the spectrum.	Dissolved sulfides in natural water samples	[34]
	Zinc capture—methylene blue method	Zinc-trapped sulfides are acidified and reacted with DMPD/Fe^3+^ to yield methylene blue (670 nm); LC-MS can be coupled for higher sensitivity.	Water samples, biological samples	[29]
Chromatography	Gas chromatography coupled with a flame photometric detector (GC-FPD)	Acid-labile sulfur (acid) and sulfane sulfur (cyanide) are detected by GC-FPD; alternatively, oxidation to sulfate/thiosulfate permits IC-electrochemical detection.	Rat liver and cardiac tissue samples	[35]
	Mobile gas dialysis chromatography	Samples are treated with HCl to release H_2_S, which is trapped in NaOH, concentrated, and detected by IC using a silver electrode for electrochemical detection.	Human and rat brain tissues	[36]
	Gas chromatography coupled with a sulfur chemiluminescence detector (GC-SCD)	Samples are injected onto a Teflon column (Chromosil 330) with nitrogen carrier, detected by chemiluminescence, and quantified by peak area.	Mouse tissues, human blood	[37]
	Gas chromatography with silver particle trapping	Ag particles adsorb H_2_S to Ag_2_S; release with thiourea/H_2_SO_4_ and GC; differentiates acid-labile from bound sulfur.	Mouse blood, rat brain and tissue homogenate	[38]
	High-performance liquid chromatography (HPLC)	Zinc-trapped analyte derivatized to methylene blue, analyzed by RP-HPLC-visible; or pre-column fluorescent derivatization with fluorescence detection.	Cow brain; Human red blood cells	[39]
	Liquid chromatography–tandem mass spectrometry (LC-MS/MS)	CBS/CSE-generated H_2_S byproducts cystathionine, lanthionine, and homolanthionine (thioethers) were measured by MRM.	Human plasma and serum	[40]
	HPLC-DAD method based on MMB or MBB	MBB alkylates H_2_S/HS^−^ to UV/fluorescent SPB/SDB for HPLC detection.	Plasma, rat tissues	[41]
Fluorescence probe method	Benzodisulfide cyclopentanone probe	Bis-electrophilic center reacts with H_2_S to fluorescence; product also MS-analyzable.	Bovine plasma	[42]
	Azide-reduced probe	H_2_S reduces azide to amine with fluorescence; rapid, no pretreatment.	Aqueous solution, bovine serum, mouse blood	[43]
	Chemiluminescence based on luminol	Samples mixed with CLSS-1/2, light recorded, H_2_S quantified by standard curve.	Cell lysis solution or culture medium	[44]
	Nitro-reduced fluorescent probe	NMO probe incubated with sample in PBS (pH 7.4), measure fluorescence at 550 nm (ex 430 nm); for cell imaging, co-incubate with HEK293T and view under fluorescence microscope.	HEK293T cells	[45]
	Acetyl benzocoumarin fluorophore	H_2_S reacts with probe’s α,β-unsaturated carbonyl via tandem Michael/aldol to turn weak to strong red fluorescence (λem 628 nm). 900 nm two-photon excitation gives red emission, avoiding autofluorescence.	Saliva, phosphate-buffered solution	[46]
	H_2_S-responsive near-infrared fluorescent probe	HT probe in DMSO/H_2_O with sample: monitor A ↓ 535, new peak 700 nm; record F ↑ 785 (ex700). For cell imaging, co-incubate and view by fluorescence microscopy.	DMSO/H_2_O solution, A549 cells, nude mice	[47]
	Chlorinated coumarin malononitrile fluorescence probe	H_2_S replaces Cl, then intramolecular addition forms cyclic imine coumarin (1b), blocking ICT/PET, enhancing fluorescence (λex/em 515/564 nm).	MCF-7 cells	[48]
Sulfide-specific ion-selective electrodes (ISE)	Direct potential method	Alkaline conversion of sulfides to S^2−^, measure electrode potential; protein interference may occur, so use with caution.	Bovine serum albumin (BSA) and buffer solution	[49]
Polarographic electrodes	Polarographic hydrogen sulfide sensor	H_2_S concentration was recorded real-time in a respirometer chamber using an H_2_S-permeable membrane and anode/cathode system.	Rat aortic smooth muscle cells and intact thoracic aorta	[50]
	Amperometric microsensor method	H_2_S-selective sensors (e.g., TBR4100) directly measure H_2_S in samples electrochemically.	Serum, tissue homogenate, kidney sections, culture supernatant	[51]

**Table 2:** Enzyme activity detection methods.

Enzyme Name	Main Organizational Expression	Activity Detection Method	Reference
CSE	Liver, kidneys, thoracic aorta, ileum, portal vein, islets of pancreas, placenta	CSE produces H_2_S from L-cysteine; H_2_S reacts with sulfite to thiosulfate, then with DTNB to yellow product (412 nm). Absorbance increase reflects H_2_S production and CSE activity.	[52]
CBS	Brain, liver, kidney, pancreas	Isotope serine -LC-MS/MS; H_2_S selective electrode/lead-acetic acid method	[40]
3-MST	Liver, kidney, heart, lung, thymus, brain, red blood cells, vascular endothelium	Coupled CAT–MST reaction system; real-time monitoring by fluorescent probe	[53]

## Data Availability

No new data were created or analyzed in this study. Data sharing is not applicable.

## Abbreviations

The following abbreviations are used in this manuscript.

NF-κB	nuclear factor κB
(TLR4)	Toll-like receptor 4
PI3K	phosphoinositide 3-kinase
Akt	protein kinase B
MAPKs	mitogen-activated protein kinases
STAT	signal transducer and activator of transcription
Nrf2	nuclear factor erythroid 2-related factor 2
SIRTs	sirtuins
NLRP3	NOD-like receptor protein 3
TNF-α	tumor necrosis factor alpha
ILs	interleukins
TRAF2	TNF receptor-associated factor 2
TMAO	trimethylamine N-oxide
CNTF	ciliary neurotrophic factor
MBP	myelin basic protein
Olig2	oligodendrocyte transcription factor 2
DADS	diallyl disulfide
SAM	S-adenosyl L-methionine
OA	osteoarthritis
MCP-1	monocyte chemoattractant protein 1
MIF	migration inhibition factor
